# Spatiotemporal Dynamics of *Vibrio* Communities and Abundance in Dongshan Bay, South of China

**DOI:** 10.3389/fmicb.2020.575287

**Published:** 2020-11-26

**Authors:** Wei Xu, LinFeng Gong, Shuai Yang, Yuanhao Gao, Xiaowan Ma, Limei Xu, Haisheng Chen, Zhuhua Luo

**Affiliations:** ^1^Key Laboratory of Marine Biogenetic Resources, Third Institute of Oceanography, Ministry of Natural Resources, Xiamen, China; ^2^Fourth Institute of Oceanography, Ministry of Natural Resources, Beihai, China; ^3^Fishery Technology Promotion Station of Dongshan, Zhangzhou, China; ^4^Co-Innovation Center of Jiangsu Marine Bio-industry Technology, Jiangsu Ocean University, Lianyungang, China; ^5^School of Marine Sciences, Nanjing University of Information Science & Technology, Nanjing, China

**Keywords:** seasonal pattern, aquaculture activity, seawater, *Vibrio* diversity, ecological distribution

## Abstract

The *Vibrio* genus inhabit estuarine and marine ecosystem throughout the world and can cause severe infections in humans and animals. Previous studies have demonstrated the dynamics of *Vibrio* at both community and population levels and assessed the close relationship between environmental factors and *Vibrio* diversity and abundance, such as temperature, salinity, and dissolved oxygen. It is also generally believed that aquaculture is the fastest-growing food sector, which is also applying great environmental impacts on microbial communities in aquatic ecosystems. However, our understanding of the spatiotemporal quantification of *Vibrio* throughout the four seasons in the aquaculture zone and response to environmental factors remains poor. To explore the spatiotemporal distribution and abundance of the *Vibrio* community with their related environmental factors and detect the relationships among them, we collected 10 seawater sites spanning four seasons across the whole year in Dongshan Bay for investigating the *Vibrio* community dynamics. Marked differences in diversity and abundance of the *Vibrio* community were observed between seasons, which were mainly driven by temperature, dissolved oxygen, nitrate, and nitrite. qPCR analysis showed that *Vibrio* abundance was most abundant in the summer (5.37 × 10^6^ copies/L), compared with the autumn (4.58 × 10^6^ copies/L), spring (1.18 × 10^6^ copies/L), and winter (1.55 × 10^4^ copies/L). A total of 22 *Vibrio* operational taxonomic units (OTUs) and 28 species were identified by universal bacteria 16S rRNA gene and cultivation methods, with *Vibrio fortis* the dominant in these aquaculture areas. To summarize, our present study is one of the few studies to research the occurrence of *Vibrio* in marine aquaculture of South China, and the results indicate that *Vibrio* are widely distributed in aquaculture environment and that a further risk assessment is needed to be conducted.

## Introduction

Currently, more than 47% of the global fish production are harvested from aquaculture, and China is the world’s largest producer of aquaculture-based foods, representing ca. 60% of global production; and marine aquaculture accounts for about 6% of our total domestic food supply (Food and Agriculture Organization of the United Nations [FAO], and The State of World Fisheries and Aquaculture [WFA], 2016). The substantial supply of nutrients can modify the local physicochemical environment and alter the microbial community structure in the aquaculture area (Dowle et al., 2015). The total bacterial populations mediate crucial biogeochemical processes in aquatic ecosystems that have been studied for a long time (Hall et al., 2008; Fonte et al., 2013; Zhu et al., 2019). However, seasonal changes affect the microbial community composition, and biomass is often attributed to responses to changes in environmental conditions driven by seasonal climate cycles (Ward et al., 2017).

The genus *Vibrio* is a group of gram-negative rods belonging to the Gammaproteobacteria with facultative fermentative metabolisms. This genus is highly heterogeneous and has abundant members of native microbial assemblages in a great variety of aquatic environment (Thompson et al., 2004; Zhang X.H. et al., 2018). More than 130 *Vibrio* species have been described to date; several *Vibrio* spp. are well known as a causative agent of foodborne-related illnesses (e.g., foodborne gastroenteritis), which can have a huge impact on public health and management (Pruzzo et al., 2005; Lee and Raghunath, 2018; Zhang X.H. et al., 2018). Most earlier studies on the *Vibrio* diversity are carried out by cultivation-dependent approaches (Baffone et al., 2006; Hsieh et al., 2008; Nigro et al., 2011; Amin et al., 2016; Kopprio et al., 2017; Wong et al., 2019). Previous studies estimated that the specificity of thiosulfate citrate bile salt sucrose (TCBS) medium for the genus *Vibrio* was approximately 60% (Oberbeckmann et al., 2012). Due to the rapid development of high-throughput sequencing (HTS) technology, it offers potential solution to overcome the limitation of traditional cultivation-dependent approach.

The ecological importance of *Vibrio* has drawn increasing attention in the study of the *Vibrio* dynamics in estuarine and coastal habitats worldwide (Thompson et al., 2004; Mansergh and Zehr, 2014; Lopez-Hernandez et al., 2015; Vezzulli et al., 2016; Liang et al., 2019). At a global scale, the abundance of *Vibrio* has been correlated with temperature and salinity in most marine environments (Thompson et al., 2004; Julie et al., 2010; Nigro et al., 2011; Oberbeckmann et al., 2012; Vezzulli et al., 2016; Zhang X.H. et al., 2018). Moreover, inorganic nutrients, such as dissolved organic carbon (DOC), dissolved inorganic carbon (DIC), NO_3_^–^, NO_2_^–^, and PO_4_^3–^, which can be directly utilized by *Vibrio* for metabolism, also exert significant influences on *Vibrio* abundance, motility, and community composition (Thompson and Polz, 2006; Kopprio et al., 2017; Liang et al., 2019). Although numerous and varying environmental parameters increase the complexity and necessity of studying the ecology of *Vibrio* in different sea areas, comprehensive investigations are necessary to define the relationship between the environment factor and *Vibrio* dynamics, especially comparing the effects of seasonality and aquaculture activity on the assembly processes of *Vibrio* communities.

Fujian Province is one of the most important aquaculture areas in South China, and the offshore water quality there also severely impacted human activities due to industry, agriculture, and a dense human-made population. The Dongshan Bay is located in the southwest of the Taiwan Strait, south of Fujian Province, China. Importantly, in recent years, increasing coastal water pollution has emerged in Dongshan Bay, which was mostly caused by human activities including increasing discharge of aquaculture, and industrial and wastewater effluent in offshore areas, which has been addressed by a previous study (Zhang W. et al., 2018). Although we know little about the large-scale distribution patterns of *Vibrio* in marine aquaculture ecosystems, several human pathogenic species, including *Vibrio cholerae*, *Vibrio parahaemolyticus*, and *Vibrio vulnificus*, are common in marine mammals, fish, and shellfish. Especially during the warm summer months, bioaccumulation in oysters and other filter feeders transmitted to humans via contaminated water or seafood, which is one of the main causes of foodborne diseases worldwide, has been widely described (Jesser and Noble, 2018). Comparing zones and their respective *Vibrio* diversity and abundance provides an excellent opportunity to detect changes in *Vibrio* communities across both seasons and aquaculture influence. However, the current information about comprehensive spatiotemporal distributions and environmental dependencies of *Vibrio* communities and abundance in these aquaculture ecosystems is still limited. The primary goal of our study was to understand the effects of seasonality and their intensity of aquaculture activities on the diversity and composition of *Vibrio* communities, which provided a valuable opportunity to demonstrate the influences of typical seasonal change on biogeochemical cycles in such systems. To solve this problem, especially for Dongshan Bay, in this study, we used HTS and qPCR analyses of the 16S rRNA gene to compare the effects of seasonality and aquaculture activity on the diversity, composition, and assembly of *Vibrio* communities in Dongshan Bay. At the same time, we evaluated the key physicochemical variables that influence the distribution of the *Vibrio* community.

## Materials and Methods

### Sample Collection and Site Description

**FIGURE 1:**
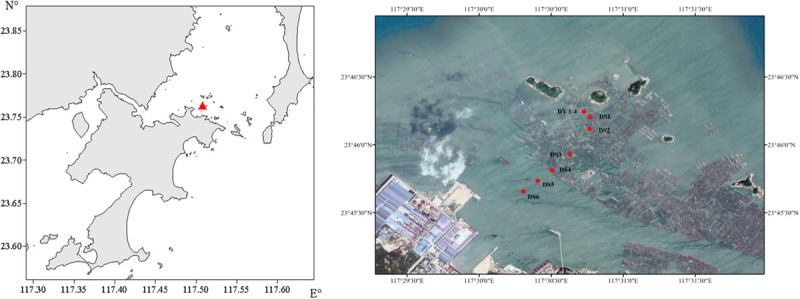
Sample area **(left)** and sampling stations **(right)**.

Ten different sampling sites were investigated around Dongshan Bay (∼24°N, ∼118°E; Figure 1). Firstly, we established three sample survey within three different zones at the Dongshan Bay [each zone differs in terms of aquaculture intensity, fish farm (FF), water channel of farm (FW), and control zone (CN)] and four sites in the fish farm (DY1–4). In Figure 1, DY represents the four sites of DY1–4 because they have the same latitude and longitude, three sites in the water channel of farm zone (DS1–4), and the remaining three sites in the control zone (DS5–6).

Surface seawater at ∼1-m depth was collected with 5-L sterile bottles at the 10 sampling sites. A total of 40 water samples were collected on January 25 (winter), April 13 (spring), July 14 (summer), and October 19, 2019 (autumn) (details are given in Supplementary Table S1). About 1 L of seawater was filtered through 0.22-μm filter units (GTBP04700, Millipore), and the resulting filters were stored at −80°C until DNA extraction. Meanwhile, 500 ml of each seawater sample was collected by a sterile bottle for nutrient analysis, and the other 50 ml of water was collected in sterile vials for *Vibrio* isolation. Parameters such as temperature, pH, salinity, and dissolved oxygen (DO) were measured *in situ* with a multiparameter water quality checker (Horiba U-50, Horiba Co., Kyoto, Japan) at each sampling site at the time of sampling. The chemical indicators of water environment, including phosphate (PO_4_^3–^) (standard method: GB/T12763.4–2007), nitrite (NO_2_^–^) (standard method: GB/T12763.4–2007), ammonia nitrogen (NH_4_^+^-N) (standard method: GB/T12763.4–2007), and nitrate (NO_3_^–^) (standard method: GB/T12763.4–2007), were measured following the corresponding standard methods. The environmental factors are expressed as mean ± standard deviation, and differences among groups were evaluated by one-way analysis of variance (ANOVA) using SPSS 16.0 software (IBM, SPSS, Chicago, United States).

### *Vibrio* Isolation and 16S rRNA Identification

All the seawater samples were immediately placed in sterile coolers, then transferred to the microbial laboratory, and processed for molecular analysis within 5 h. Seawater corresponding to marine aquaculture areas was diluted with sterile seawater, and 200 μl of the appropriate decimal dilutions was plated on TCBS agar (Oxoid) both directly and after a 10-fold dilution. At room temperature (25 ± 3°C) after 48-h incubation, retrieve all the culturable heterotrophic bacteria able to grow on TCBS. From each plate, isolated colony types of each different size and morphology were picked out and streaked onto 2216E Agar to obtain pure cultures for molecular identification. The genomic DNA of each isolate was prepared following the Bacterial DNA Kit. Molecular identification at the species level was obtained by sequencing of 16S rRNA by amplifying the specific primer pair 27F (5′-TGGCTCAGATTGAACGCTGGCGG-3′) and 1492R (5′-TACCTTGTTACGACTTCACC-3′) (Hayashi et al., 2002). PCRs were performed as follows: initial denaturation at 94°C for 3 min, followed by 35 cycles of denaturation at 94°C for 1 min, annealing for 1 min at 55°C and extension at 72°C for 1.50 min, and the final elongation step at 72°C for 5 min. PCR products were sequenced using 27F/1492R primers on an ABI 3730XL automatic sequencer by Majorbio Sequencing Service (Shanghai). The sequences of all isolates was compared with that of closely related reference strains using the EzTaxon-e server. Multiple sequence alignments were performed with CLUSTAL W. The nucleotide sequence of almost the entire 16S rRNA gene of *Vibrio* sp. isolates was deposited at GenBank with the following accession numbers: MT269611–MT269621 (January 2019), MT269622–MT269633 (April 2019), MT269634–MT26941 (July 2019), and MT269602–MT269610 (October 2019).

### DNA Extraction, PCR Amplification, and Illumina Sequencing

Genomic DNA was extracted from each filter by using the FastDNA Spin Kit for Soil and a FastPrep-24 Instrument (MP Biomedicals, United States) according to the manufacturer’s protocol. PCR amplification of V3–V4 variable regions of the 16S rRNA genes was performed using the universal primers 338F (5′-ACTCCTACGGGAGGCAGCA-3′) and 806R (5′-GGACTACHVGGGTWTCTAAT-3′) (Guo et al., 2017). Each DNA sample was PCR-amplified individually with three technical replicates. Amplicons after index added and equally pooled were then sent for the high-throughput sequencing by using Illumina HiSeq 2500 Platform (PE250) at Majorbio Bio-Pharm Technology, Shanghai, China. The raw sequencing reads have been submitted to the Sequence Read Archive (SRA) database with accession number SRR11929868–SRR11929907. Raw FASTQ files were processed and quality-filtered using the FLASH and Trimmomatic software. Operational taxonomic units (OTUs) were defined as clusters sharing 97% sequence similarity cutoff using USEARCH software (version 7.1), and chimeric sequences were identified and removed using UCHIME software (version 4.2). The Chao index and Shannon index were calculated based on OTU dilution curve analysis. The taxonomy of each representative OTU 16S rRNA gene sequence was analyzed by using the RDP Classifier (http://rdp.cme.msu.edu/) against the SILVA (SSU132) database using a confidence threshold of 70%. The quality-checked data were then analyzed on the free online platform of the Shanghai Majorbio I-Sanger Cloud Platform (www.i-sanger.com) based on QIIME 1.9.0 pipeline to obtain parameters of alpha and beta diversity indices.

### Construction of Quantitative PCR Standard

Detection of the abundance of total bacteria and *Vibrio* spp., the primer set A-967F/B-1046R, and *Vibrio* specific primers 567F/680R were used to amplify the 16S rRNA gene in the aquaculture surface water samples, carried out as described in the previous study (Liang et al., 2019). Briefly, PCR standard curves were prepared from genomic DNA extracted from *Escherichia coli* and *Vibrio alginolyticus* (Marine Culture Collection of China strain, MCCC1A06468 and MCCC1A07292), which are equivalent to 10^2^–10^8^ gene copies per reaction. The environmental DNA and control DNA were used as DNA templates in real-time PCRs, which were carried out on LightCycler 480II Real-Time PCR system (Roche Life Science, Swiss). The real-time PCR was performed with a final volume of 25 μl, including 12.5 μl of 2 × SYBR Green Real-time PCR Master Mix, 0.2 μM for each forward and reverse primers (for bacteria) or 0.4 μM for each forward and reverse primers (for *Vibrio*), 2 μl of diluted (1:10) template DNA, and double-distilled water added up to 25 μl. The real-time PCR was run with the following program: 95°C for 10 min, followed by amplification (45 cycles of 15 s at 95°C and 1 min at 60°C) and a melting curve (5 s at 95°C, 60 s at 57°C or 65°C, and increasing temperature from 65°C to 98°C at 0.11°C⋅s^–1^ with 5 fluorescence acquisitions⋅s^–1^). In the qPCR analysis, each reaction was performed with three repeats, standard curves were run with every plate and ddH_2_O as templates were used as a no-template control (NTC). The LightCycler480 software was used for statistical analyses, and final abundance is reported as gene copies/L of seawater sampled.

### Statistical Analysis

All statistical tests were considered significantly at *p* < 0.05. Seasonal differences in environmental parameters were evaluated using the non-parametric Mann–Whitney test. Abundances of *Vibrio* and bacteria were log 10^(x+1)^ transformed before the analyses. In cases where the abundance of some samples was below the detection limit of the available method, a value of zero was assigned before logarithmic conversion. Variations in *Vibrio* abundance between seasons were tested for significance using the Mann–Whitney test. Spearman’s rank correlation was calculated to determine the relationship between environmental factors and *Vibrio* abundance. All the above analyses were performed using SPSS statistical software version 16.0.

## Results

### Seasonal and Spatial Variation in Environmental Variables

**FIGURE 2:**
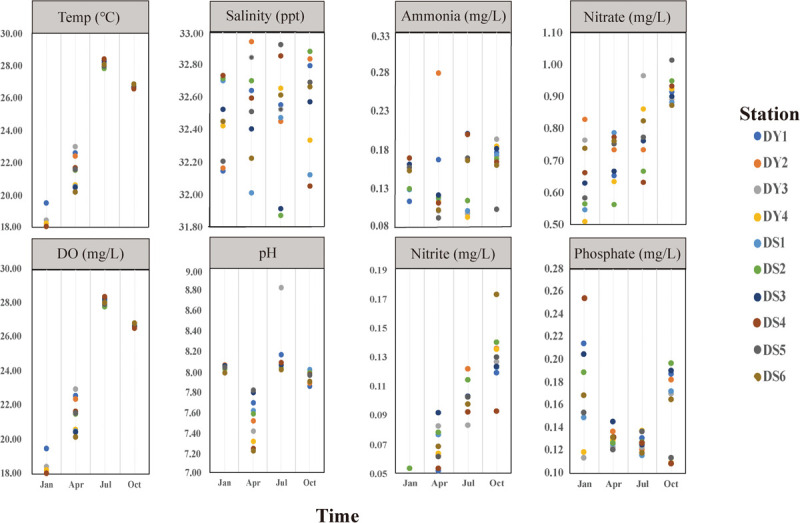
Physicochemical parameters of sampling station variables from January to October 2019 in Dongshan Bay, Fujian, China.

The environmental parameters of the seawater samples we tested, including water temperature, salinity, DO, ammonia NH_4_^+^, nitrite NO_2_^–^, nitrate NO_3_^–^, and phosphate PO_4_^3–^, and detailed physicochemical parameters of water quality for 10 stations are shown in Figure 2 (Table S1). All the samples were divided into four clusters based on seasonal patterns and physicochemical parameters, which are summarized in Table 1. Water temperature and nitrite NO_2_^–^ concentrations varied significantly between seasons, with temperature varying from 18.05 ± 0.31°C (mean ± standard deviation; winter) to 28.07 ± 0.22°C (summer), while and nitrite NO_2_^–^ ranged from 0.07 ± 0.01 (spring) to 0.92 ± 0.04 (autumn). In addition, generally, DO decreased from spring to autumn, whereas nitrate NO_3_^–^ showed opposite trends (Table 1). Our clustering analysis of the normalized environmental parameters using the average cluster method indicated that the environmental variable clustering was strongly affected by seasonal pattern, with values taken in winter and spring distinctly different from those of the other two seasons (Supplementary Figure S1).

**TABLE 1:** Summary of physicochemical parameters in four seasons.

**Physicochemical parameter**	**Value**			
	**Winter (Jan)**	**Spring (Apr)**	**Summer (Jul)**	**Autumn (Oct)**
Temp(°C)	18.052 ± 0.315	21.434 ± 0.991	28.068 ± 0.220	26.675 ± 0.099
pH	8.036 ± 0.024	7.457 ± 0.209	8.123 ± 0.249	7.947 ± 0.052
DO (mg/L)	7.926 ± 1.020	7.489 ± 0.500	4.997 ± 0.460	5.153 ± 0.387
Salinity(ppt)	32.445 ± 0.226	32.507 ± 0.295	32.480 ± 0.347	32.561 ± 0.294
NH_4_^+^(mg/L)	0.145 ± 0.019	0.121 ± 0.028	0.132 ± 0.042	0.166 ± 0.008
PO_4_^3–^(mg/L)	0.168 ± 0.044	0.130 ± 0.007	0.126 ± 0.007	0.160 ± 0.035
NO_3_^–^(mg/L)	0.665 ± 0.118	0.710 ± 0.076	0.786 ± 0.096	0.918 ± 0.041
NO_2_^–^(mg/L)	0.0179 ± 0.016	0.069 ± 0.013	0.101 ± 0.011	0.129 ± 0.020

### Spatiotemporal Variation in Abundance of *Vibrio*

**FIGURE 3:**
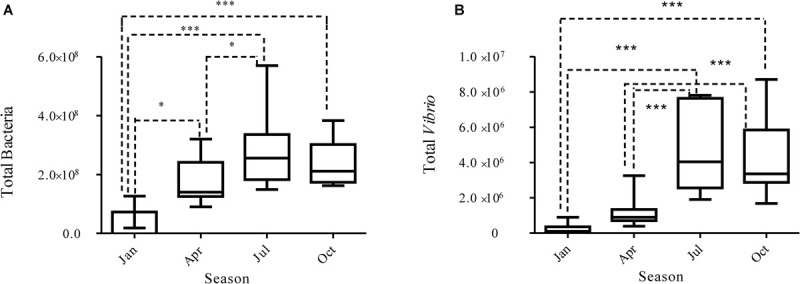
*Vibrio* **(B)** and bacterial **(A)** abundance determined by qPCR in different seasons.

The *Vibrio* community-specific qPCR assays were performed to quantify the abundance of *Vibrio* in all samples, which showed an obvious seasonal pattern (Figure 3). *Vibrio* were most abundant across the 10 sites in summer, averaged 5.37 × 10^6^ copies/L, significantly higher than those in winter (Mann–Whitney test, *p* < 0.01), with a mean value of 1.55 × 10^5^ copies/L. Significant seasonal differences in bacterial abundance were also observed (Mann–Whitney test, *p* < 0.001). We compared the *Vibrio* abundance in different samples; the fold between the highest (JulDY4) and lowest (JanDS2) is 115. We also compared the bacteria abundance in different samples; the ratio between the highest (OctDS2) and lowest (JanDY4) is 5,614. Moreover, *Vibrio* also varied in abundance between different sample areas (Kruskal–Wallis, *p* < 0.05), the abundance of *Vibrio* was higher in DS1 (farm water channel) than in DS6 (control zone) (Kruskal–Wallis, *p* < 0.05), whereas similar variations were also observed in other seasons (Supplementary Figure S2).

In winter, the *Vibrio* abundance showed positive correlation with pH (Spearman correlation, r = 0.7354, *p* < 0.05) and DO (r = 0.774, *p* < 0.05), while only nitrate (r = 0.414, *p* < 0.05) showed positive correlation with *Vibrio* abundance in summer. Moreover, there was no significant correlation between *Vibrio* abundance and biogeochemical parameters in spring and autumn. Across the entire data, the abundance of *Vibrio* spp. not only positively correlated with temperature (r = 0.799, *p* < 0.01), nitrate NO_3_^–^ (r = 0.672, *p* < 0.01), and nitrite NO_2_^–^ (r = 0.771, *p* < 0.01) but also negatively correlated with DO (r = -0.702, *p* < 0.01) (Table 2).

**TABLE 2:** Spearman rank correlations of qPCR results of *Vibrio* with environmental variables between four seasons.

**Environmental**	**Winter**				**Spring Summer**						**Autumn**			**Four seasons**		
	
**variable**	**r**	**p**	**N**	**r**		**p**	**N**	**r**	**p**	**N**	**r**	**p**	**N**	**r**	**p**	**N**
Temperature	0.248	0.489	10	0.243		0.498	10	−0.455	0.187	10	−0.311	0.382	10	**0.799**	**6.36E-10**	**40**
pH	**0.735**	**0.015**	**10**	0.426		0.220	10	0.160	0.659	10	0.415	0.233	10	0.036	0.827	40
DO	**0.774**	**0.009**	**10**	0.170		0.638	10	0.152	0.676	10	0.201	0.578	10	**-0.702**	**4.40E-07**	**40**
Salinity	−0.043	0.907	10	-0.049		0.894	10	0.358	0.310	10	0.134	0.713	10	0.164	0.313	40
NH_4_^+^	0.246	0.493	10	0.267		0.455	10	−0.588	0.074	10	−0.188	0.603	10	0.077	0.638	40
PO_4_^3–^	0.365	0.300	10	-0.226		0.531	10	0.018	0.960	10	0.541	0.106	10	−0.119	0.466	40
NO_3_^–^	0.309	0.385	10	0.0427		0.907	10	**0.693**	**0.026**	**10**	0.382	0.276	10	**0.672**	**2.03E-06**	**40**
NO_2_^–^	−0.067	0.854	10	0.128		0.725	10	−0.432	0.213	10	−0.109	0.763	10	**0.771**	**5.71E-09**	**40**

*Significant correlations (*P* < 0.05) were showed in bold. Correlation (r) and significance (*P*) values are shown.*

### Seasonal Succession of *Vibrio* Composition by 16S rRNA Sequencing

**FIGURE 4:**
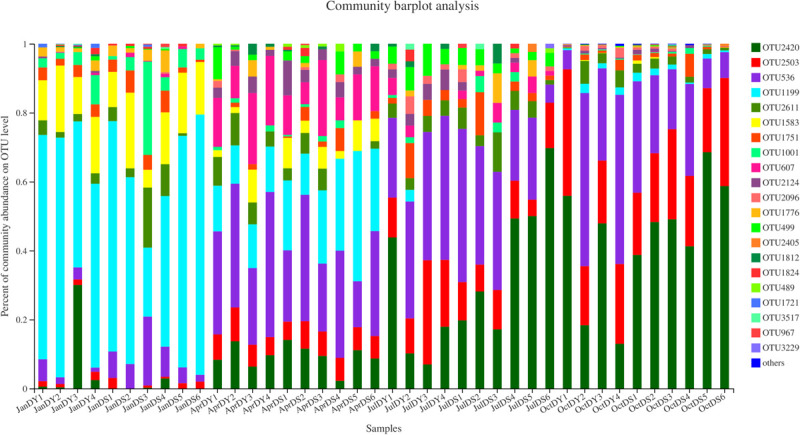
*Vibrio* community compositions at the species level across all samples.

*Vibrio* diversity was assessed via the amplification and sequencing of the V3–V4 region of the 16S rRNA gene. In total, 1,539,256 clean reads were obtained through Illumina sequencing after quality control, ranging from 19,816 to 62,826 reads, individually. The total sequences yielded 3,057 OTUs at a 97% sequence similarity level. Of these, a total of 22,786 reads and 22 OTUs reads were selected, which were classified into the genus of *Vibrio* (Figure 4 and Supplementary Table S2). The mean (± SD) relative abundance of *Vibrio* spp. 16S rRNA gene sequence reads in water samples from summer and autumn of those stations was 0.33% (±0.12%) and 9.14% (±8.48%), respectively. The highest relative *Vibrio* abundance of total bacteria in the water samples (12.83%) was measured in DS6 at autumn, where station DS6 was located at the control zone.

Representative sequences of each OTU were compared against the EzBioCloud and National Center for Biotechnology Information (NCBI) database to determine their taxonomic status, which clustered with *Vibrio aestivus*, *Vibrio caribbeanicus*, *Vibrio cortegadensis*, *Vibrio echinoideorum*, *Vibrio fortis*, *Vibrio furnissii*, *Vibrio gigantis*, *Vibrio hepatarius*, *Vibrio harveyi*, *Vibrio maritimus*, *Vibrio mimicus*, *Vibrio neptunius*, *Vibrio ponticus*, *Vibrio renipiscarius*, *Vibrio rotiferianus*, *Vibrio zhanjiangensis*, and two unclassified *Vibrio* spp. (Supplementary Table S3). Our samples did not contain sequences clustering with the common pathogens *Vibrio alginolyticus*, *Vibrio cholerae*, *Vibrio coralliilyticus*, *Vibrio parahaemolyticus*, *Vibrio pectenicida*, *Vibrio splendidus*, *Vibrio tubiashii*, and *Vibrio vulnificus.* There were no significant differences (*p* > 0.05) in *Vibrio* diversity with respect to the different seasons. There are 14 OTUs detected from all the four seasons, while only one, two, and two specific OTUs were specifically found in spring, autumn, and winter samples (Supplementary Figure S3). The most dominant species was *V. fortis* OTU2420, occupying 44.23% of all sequences, followed by *Vibrio brasiliensis* OTU2503, *V. harveyi* OTU536, and *V. gigantis* OTU1199, which jointly accounted for 47.77% of all sequences (Supplementary Figure S4). OTU1812 was not resolved at the species level and showed the highest 16S rRNA gene similarity to *Vibrio* sp. strain r32 (NCBI Taxonomy ID AB470935) (93.01%), which may represent a novel *Vibrio* species.

**FIGURE 5:**
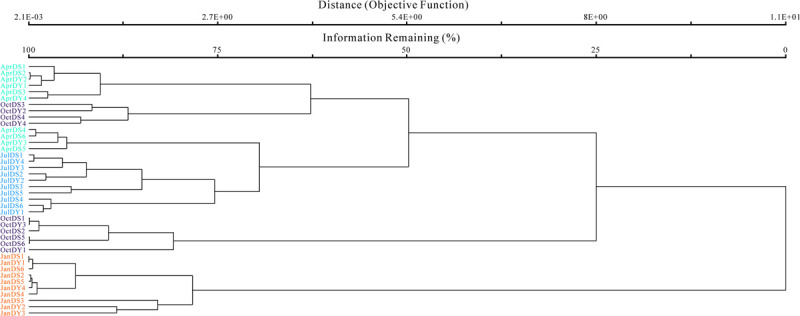
Weighted Bray–Curtis analysis was conducted at the operational taxonomic unit (OTU) level.

To illustrate the diversity pattern of *Vibrio* spp. across all samples, the OTU-level weighted FastUnifrac analysis was conducted (Figure 5), which showed that the samples clustered together into two larger clusters and were separated into four clades according to seasonal pattern, with the exception that OctDS3, OctDY2, OctDS3, and OctDY2 are clustered with April samples showing that there is little *Vibrio* difference between spring and several autumn samples. In the surface water, the total average number of *Vibrio* OTUs in autumn (14.2 ± 2.4) is significantly higher than in summer (10 ± 2.1). The unweighted FastUnifrac analysis showed that there was no significant difference between the different terms of aquaculture intensity. After comparison of the specific *Vibrio*-related OTUs in different zones, it appeared that there are 20 OTUs obtained from all the three zones, while water channel samples recovered only one specific OTU (Supplementary Figure S5). These results revealed that the seasonal variations were more pronounced than the spatial variations.

**FIGURE 6:**
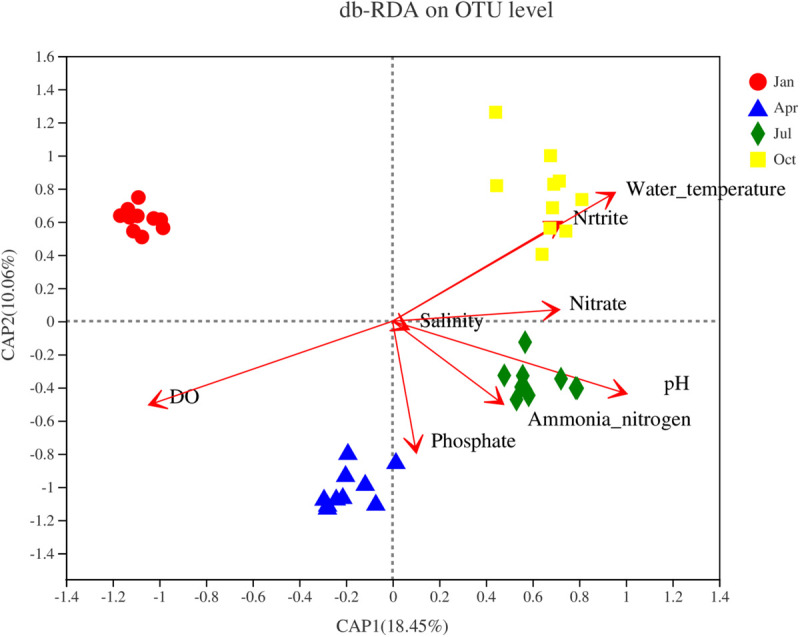
Distance-based redundancy analysis (db-RDA) illustrating the relationship between the *Vibrio* community at the operational taxonomic unit (OTU) level and environmental variables.

The relationship between environmental and spatial factors and *Vibrio* community components at the OTU level was investigated by the Mantel test and distance-based redundancy analyses (db-RDAs) (Figure 6). The first axis explained 18.45% of the total variance, whereas the second axis explained 10.06%. Water temperature (R^2^ = 0.88, *p* < 0.001), pH (R^2^ = 0.60, *p* < 0.001), phosphate PO_4_^3–^ (R^2^ = 0.30, *p* < 0.001), nitrate NO_3_^–^ (R^2^ = 0.28, *p* < 0.001), and nitrite NO_2_^–^ (R^2^ = 0.28, *p* < 0.001) positively correlated with RDA1, while DO (R^2^ = 0.89, *p* < 0.001) was negatively correlated with RDA1. No significant correlation was observed between salinity and NH_4_^+^ ammonia nitrogen (*p* > 0.05).

The Spearman correlations between the 22 *Vibrio* OTU and environmental factors were calculated and summarized in Table 3, with nine OTUs significantly correlated with four environmental factors. The most abundant species *V. fortis* (OTU2420) had significant correlations with DO (r = -0.602, *p* = 0.008), nitrate NO_3_^–^ (r = -0.583, *p* = 0.031), and nitrite NO_2_^–^ (r = -0.739, *p* = 0.00002). *V. gigantis* (OTU1199), which is the dominant group in spring, was positively related to DO (r = 0.682, *p* = 0.0005) and negatively related to nitrite NO_2_^–^ (r = -0.573, *p* = 0.044) and temperature (r = -0.832, *p* = 0.0000000123). In addition, of the two specific *Vibrio* OTUs in autumn, *V. fortis* (OTU2405) was positively collected with nitrite NO_2_^–^ (r = 0.606, *p* = 0.014), while *V. maritimus* (OTU2461) was positively collected with nitrate NO_3_^–^ (r = 0.670, *p* = 0.0009) and nitrite NO_2_^–^ (r = 0.628, *p* = 0.006).

**TABLE 3:** Correlations between percentage composition of OTU and environmental factors.

**OTU**	**Correlation coefficient^1^**							
	**Temperature**	**pH**	**DO**	**Salinity**	**NH_4_^+^**	**PO_4_^3–^**	**NO_3_^–^**	**NO_2_^–^**
*Vibrio caribbeanicus* OTU607								
*Vibrio fortis* OTU2405								0.606
*Vibrio mimicus* OTU3229								
*Vibrio* sp. OTU1812								
*Vibrio maritimus* OTU967								
Vibrio fortis OTU2420			**−0.620**				**0.582**	**0.739**
*Vibrio zhanjiangensis* OTU1721								
*Vibrio ponticus* OTU1751								
*Vibrio maritimus* OTU2461							**0.670**	**0.628**
*Vibrio natriegens* OTU536								**0.619**
*Vibrio renipiscarius* OTU1001	−0.578							
*Vibrio aestivus* OTU1824								
*Vibrio echinoideorum* OTU1583	**−0.836**		**0.709**					**−0.659**
*Vibrio* sp. OTU3517								
*Vibrio natriegens* OTU2611								
*Vibrio cortegadensis* OTU1776								
*Vibrio rotiferianus* OTU2096								**0.662**
*Vibrio hepatarius* OTU2124								
*Vibrio pomeroyi* OTU1199	**−0.832**		**0.682**					−0.572
*Vibrio caribbeanicus* OTU499								
*Vibrio neptunius* OTU2503							**0.638**	**0.693**
*Vibrio furnissii* OTU489								

*Only significant correlations (*P* < 0.05) are shown. −, negative; boldface, *P* < 0.01; lightface, *P* < 0.05.*

### Identification of *Vibrio* Diversity by Cultivation Method

A total of 201 presumptive *Vibrio* spp. were isolated; most isolates were confirmed as *Vibrio* spp. via the 16S rRNA sequence. There were also a significant number of isolates (50 isolates) that were identified as *Photobacterium* spp., *Shewanella* spp., *Enterovibrio* spp., *Bacillus* spp., *Grimontia* spp., *Psychrobacter* spp., and *Shewanella* spp. A total of 28 *Vibrio* species were identified; the most species-rich season was spring, with 12 species (42 isolates). The winter harbored 11 species (39 isolates) and autumn with nine species (32 isolates). The lowest species-rich season was summer, with only eight species (38 isolates). Three *Vibrio* spp. commonly occurring in different seasons were *V. fortis*, *V. alginolyticus*, and *Vibrio hyugaensis*, while the most frequently isolated *Vibrio* sp. was *Vibrio crassostreae*. Two species (*V. parahaemolyticus* and *V. brasiliensis*) were isolated in summer, whereas four species (*Vibrio shilonii*, *Vibrio diabolicus*, *Vibrio xuii*, and *V. ponticus*) were obtained only at autumn, and *Vibrio sinensis* was only detected in winter (Supplementary Table S4). Based on the Shannon diversity index, *Vibrio* community at spring (H’ = 2.64 ± 0.12) was more diverse than summer (H’ = 1.90 ± 0.23) (*p* = 0.05). To identify specific environmental factors that may shape the community structure, RDA was conducted. RDA1 explained 62.43% of the total variation, while RDA2 explained 24.72% (Supplementary Figure S6). Only nitrate NO_3_^–^ (F = 2.5, *p* < 0.05) was the most important factor that influenced the *Vibrio* diversity.

## Discussion

Dongshan Bay is a semi-enclosed bay on the southeast coast, and it is an important aquaculture base for Fujian Province. The genus *Vibrio* is frequently associated with both wild and farmed marine animals for the ability of some species as the leading cause of foodborne outbreaks (Alam et al., 2003; Lee et al., 2008; Liu et al., 2017) and is also considered a significant problem with severe economic losses in aquaculture industry worldwide (Mancuso et al., 2015). Various studies aimed at understanding the *Vibrio* biogeography patterns and environmental population temporal dynamics have focused mostly on coastal and estuarine ecosystems, which suffer less human influence (Mansergh and Zehr, 2014; Westrich et al., 2016, 2018). However, current information about comprehensive spatiotemporal distributions and environmental dependencies of *Vibrio* communities and abundance in these aquaculture ecosystems is still limited. Overall, our results characterized that *Vibrio* communities in the Dongshan Bay were strongly impacted by the local seasonality, such as the fluctuations of temperature and physiochemical characters. This finding provided insights into response to environmental change, which can potentially be used to take control of blooms by interventional strategies.

Apparent seasonal variations in microbial community structure have been shown in diverse coastal ecosystems (Kirchman et al., 2005; Gilbert et al., 2012; Lau et al., 2013; Suh et al., 2015; Wang et al., 2019). The abundance and community composition of *Vibrio* populations in marine environments are shaped by various environmental parameters, notably temperature, salinity, and DOC (Takemura et al., 2014; Siboni et al., 2016; Kopprio et al., 2017; Zhang X.H. et al., 2018). Our results demonstrated that *Vibrio* diversity and abundance typically fluctuate seasonally, that the temperature had a positive correlation with the *Vibrio* community, and that either salinity or pH had no correlation with it.

The abundance of *Vibrio* spp. also showed obvious differences between aquaculture-influenced zones within the same season, especially between the FF zone and CN zone (*p* < 0.01), which is largely due to the difference between the amount of nutrient input in the fish farm (Fig. S2). This significant difference suggested a direct correlation between the distribution of *Vibrio* with the nearby aquaculture zone, which mainly farms the *abalone*. Many previous studies have reported the impacts of aquaculture activities on the microbial biogeography targeting the total bacterial population in the coastal environment (Pereira et al.2011; Sundberg et al.2016; Prado et al., 2020), and thus, specific lineages have been often neglected. Our results suggest that a specific phylogenetic group, that is, *Vibrio*, can also exhibit a clear spatial distribution pattern within the season, which is consistent with evidence by Dowle et al. (2015) and Zeng et al. (2019), who reported that total bacterial diversity changed with distance away from aquaculture zones. Besides, bacterial 16S rRNA is two to three orders of magnitude higher than the *Vibrio-*specific 16S rRNA, except for one sample in the fish farm at winter DY3 with only two hundred thousandths. These results are consistent with the research by Thompson et al. (2004), which found that the relative abundance of *Vibrio* was generally <2% of the total bacterioplankton.

A total of 167 *Vibrio* strains (belonging to 28 species) were identified, whose species richness was also consistent with that of other studies using culture-dependent approaches and the majority of *Vibrio* spp. isolates previously reported (Tall et al., 2013; Siboni et al., 2016; Wong et al., 2019). However, compared with *Vibrio* diversity in other temperate and subtropical coastal environments in South China Sea (Li et al., 2019; Möller et al., 2020), our results showed a differently structured *Vibrio* community. The diversity of *Vibrio* was higher in winter than in summer, which is consistent with the previous study in the water column of northern Chinese marginal seas (Liang et al., 2019). But these results are in contrast to the results of a previous study in which *Vibrio* was reported to be more diverse in summer than in winter (Siboni et al., 2016). The discrepancy between this previous study and our present study may be due to both that there was an actual change in the occurrence rates and that the nutrient concentrations of aquaculture from artificially affecting were different over the different regions.

Seasonality has a complex jointly impact on many hydrological gradients and inorganic and organic nutrients, which may alter the *Vibrio* diversity, which is also the most commonly cited reason in other reports about the *Vibrio* community structure change (Zhang X.H. et al., 2018; Liang et al., 2019). Furthermore, the second and third strongest environmental parameters (salinity and pH, respectively) that correlate with *Vibrio* diversity also do not appear to be involved in the present study. Measured salinity and pH have remained changed slightly. On the other hand, DO concentration decreased from winter to autumn, which should be associated with a subsequent decrease in *Vibrio* spp. In terms of inorganic and organic nutrients, NOx and PO_4_^3–^ were found to have correlated with the change in *Vibrio* spp. There was a marked decrease in NOx in the same years as the largest increases in *Vibrio* spp. In our study, *Vibrio* spp. abundance was found to increase with concentrations of NO_2_^–^ and NO_3_^–^, which is consistent with observations in the Arabian Sea and Sydney Harbor estuary that spatial and temporal shifts in the abundance of the *Vibrio* community between seasons are primarily driven by the change of NO_3_^–^ and NO_2_^–^ (Asplund et al., 2011; Siboni et al., 2016). Similar relevant relationships between environmental factors and the composition of the *Vibrio* community were also observed with the most abundant OTU (*V. fortis* OTU2420), which comprised up to 43.2% of the community, and significantly correlated with DO, NO_2_^–^, and NO_3_^–^. These nutrition factors, including NOx and phosphate PO_4_^3–^, were found to have correlated with the change of *Vibrio* spp. Contraction; a recent study suggested that they were more likely a result of the increased bacterial population, rather than a cause of nutrition triggers (Froelich et al., 2019).

Furthermore, the frequently occurring *Vibrio* spp. in the present study were *Vibrio fortis*, *Vibrio alginolyticus*, and *Vibrio hyugaensis*, which suggested that the predominance of a specific *Vibrio* sp. seems to be site-specific as others have reported different predominant *Vibrio* species in their studies, such as *V. alginolyticus*, *Vibrio campbellii*, *Vibrio carchariae*, *Vibrio harveyi*, *Vibrio owensii*, *Vibrio parahaemolyticus*, *Vibrio rotiferianus*, and *Vibrio splendidus* (Maeda et al., 2003; Eiler et al., 2006; Ortiz-Carrillo et al., 2015; Li et al., 2019; Wong et al., 2019). The four major clinically important pathogenic *Vibrio* species, *Vibrio cholerae*, *V. parahaemolyticus*, *V. alginolyticus*, and *Vibrio vulnificus*, have been implicated in human diseases such as gastroenteritis and septicemia and even death in immunocompromised patients (Novoslavskij et al., 2016). In the present study, the occurrence rate of *V*. *alginolyticus* was much higher than that of *V*. *parahaemolyticus*, whereas *V*. *cholerae* and *V*. *vulnificus* were not isolated, but these two lost *Vibrio* spp. have been extracted from oysters *Crassostrea commercialis* farmed at estuaries along the east coast of Australia (Siboni et al., 2016). They may have been contaminated by human fecal material, and these differences may also be explained by the halophilism of the four pathogens or their low growth rates in different nutrient conditions (Wong et al., 2019; Yan et al., 2019). Moreover, other two *Vibrio* species (*Vibrio mimicus* and *Vibrio furnissii*) that may have the pathogenic potential for humans were isolated from the seawater in our study (Zhang et al., 2015), and two marine animal pathogens *V. harveyi* and *V. furnissii* were also recovered by metabarcoding (Matteucci et al., 2015).

The most predominant *Vibrio* sp. in our study is *V. fortis*, which differed from *Vibrio lentus* in the Neuse River Estuary (Jesser and Noble, 2018) and *V. campbellii*, *Vibrio caribbeanicus*, or *Vibrio atlanticus* in the water column from the Chinese marginal seas (Liang et al., 2019). Recently, the effects of water temperature were examined on the microbiome of Pacific oyster *Crassostrea gigas* by using an experiment designed to replicate the effect of a marine heat wave event, yet this study found that heat stress increased the abundance of *V. fortis* by 10-fold (Green et al., 2019), indicating that the growth of *V. fortis* has been promoted by warmer temperatures, and they may represent warm-water species.

In addition, another paradoxical and impenetrable phenomenon is that the disappearance of the summer predominant *Vibrio* species may be associated with the high diversity of *Vibrio* that was observed in winter. One reason is that the food supply for aquaculture animals has introduced more nutrients that favor primary productivity and subsequently the growth of heterotrophic bacteria, including *Vibrio* spp. Most studies on *Vibrio* diversity have an agreement that many *Vibrio* spp. enter viable but non-culturable growth states (VBNCs) when exposed to adverse growth conditions will underestimate *Vibrio* diversity, as we know that the water temperatures that increased the effect of VBNC on our *Vibrio* spp. counts were presumed to be negligible (Oliver, 2010; Vezzulli et al., 2012, 2013; Takemura et al., 2014).

After identification of OTUs in SILVA16S rRNA database, taxonomy was also reassigned against the EzBioCloud database, which will give more information about the latest taxonomy of microbial isolates and more complete taxonomic hierarchy from phylum to species for the isolates (Quast et al., 2012; Yoon et al.2017). Several OTUs annotated as *Vibrio* spp. (OTU967, OTU1721, OTU2461, OTU1824, OTU1583, OTU2611, OTU1776, OTU2096, OTU2124, OTU499, and OTU489) in SILVA were assigned to *Vibrio maritimus*, *Vibrio zhanjiangensis*, *V. maritimus*, *Vibrio aestivus*, *Vibrio echinoideorum*, *Catenococcus thiocycli*, *Vibrio cortegadensis*, *V. rotiferianus*, *Vibrio hepatarius*, *V. caribbeanicus*, *V. furnissii*, and also three OTUs (OTU607, OTU1812, and OTU3517) cannot be affiliated to species level. It was reported that universal 16S rRNA primers may have some limitations; the most important fact is that it does not contain highly variable domains that are probably required to analyze relationships of closely related organisms, which indicated that it was necessary for *Vibrio* to develop a high species diversity, such as *Vibrio*-specific 16S rRNA and *hsp60* sequencing retrieved more than did the present study (Siboni et al., 2016; Jesser and Noble, 2018; Liang et al., 2019).

## Conclusion

This study investigated the *Vibrio* communities within aquaculture zone in relation to the established environment factors based on 1-year survey. This observation demonstrated the importance of seasonal pattern in determining the *Vibrio* abundance and community composition on these three different zones, temporal-spatial assemblages on these three different zones, where significant abundances of *Vibrio* already occur, are potentially at risk of pathogenic *Vibrio* outbreaks, particularly during warm summer months. Both the abundance and community composition varied between different sea areas, with the *Vibrio* assemblages displaying a clear distance-decay pattern. In addition, *Vibrio fortis* was dominant in these aquaculture areas by universal bacteria 16S rRNA gene and cultivation methods. Our present study is one of the few studies to examine the occurrence of *Vibrio* in the marine aquaculture zone of South China, and the results indicate that *Vibrio* are widely distributed in aquaculture environment and that a further risk assessment is needed to be conducted.

## Data Availability Statement

The raw sequencing reads have been submitted to the Sequence Read Archive (SRA) database with accession number SRR11929868–SRR11929907.

## Author Contributions

WX is responsible for article writing and data analysis. LG, XM, and ZL are responsible for data analysis. SY and YG are responsible for sample collection. LX is responsible for experiment. CS is responsible for sample collection. All authors contributed to the article and approved the submitted version.

## Conflict of Interest

The authors declare that the research was conducted in the absence of any commercial or financial relationships that could be construed as a potential conflict of interest.

## Supplementary Material

Supplementary Figure 1 Clustering analysis of the normalized environmental parameters using the average cluster method.

Supplementary Figure 2 *Vibrio* abundance determined by qPCR in the three different sample areas (fish farm, water channel of farm zone and control zone). The asterisks denote significant differences between seasons. **P* ¡ 0.05, **P*P* ¡ 0.01.

Supplementary Figure 3 Venn diagram showing the OTUs that obtained and overlap among total samples across the four seasons.

Supplementary Figure 4 *Vibrio* community structure determined by dominant OTUs counted in the 40 water samples.

Supplementary Figure 5 Venn diagram showing the OTUs that obtained and overlap among total samples within three different zones.

Supplementary Figure 6 RDA biplots for four seasons based on *Vibrio* isolates data and environmental variables.

Supplementary Table 1 Environmental parameters of all sampling sites across the four seasons.

Supplementary Table 2 Observed reads and diversity estimates of *Vibrio* spp. based on 97% OTU clusters.

Supplementary Table 3 Classification of obtained 22 *Vibrio* OTUs based on three different database.

Supplementary Table 4 Classification of obtained 23 *Vibrio* species based on 16S rRNA sequences.
