# Supplementary figures and images for: Spatiotemporal Dynamics of Vibrio Communities and Abundance in Dongshan Bay, South of China
Source: Front Microbiol. 2020 Nov 26;11:575287. doi: 10.3389/fmicb.2020.575287 (PMC7726330; doi:10.3389/fmicb.2020.575287)

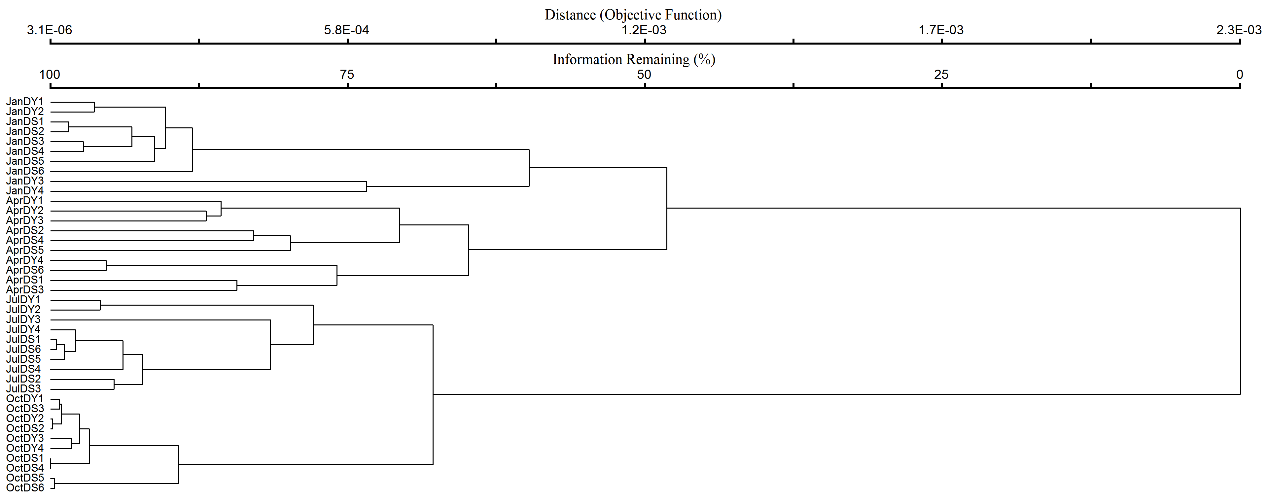

Supplement: Supplementary Figure 1 — Clustering analysis of the normalized environmental parameters using the average cluster method. [file Image_1.TIF]

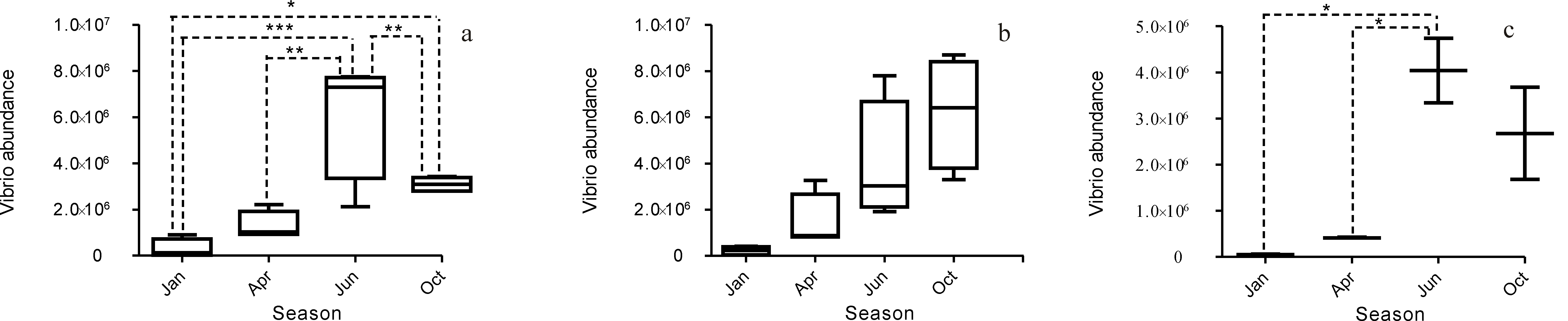

Supplement: Supplementary Figure 2 — Vibrio abundance determined by qPCR in the three different sample areas (fish farm, water channel of farm zone and control zone). The asterisks denote significant differences between seasons. *P ¡ 0.05, **PP ¡ 0.01. [file Image_2.TIF]

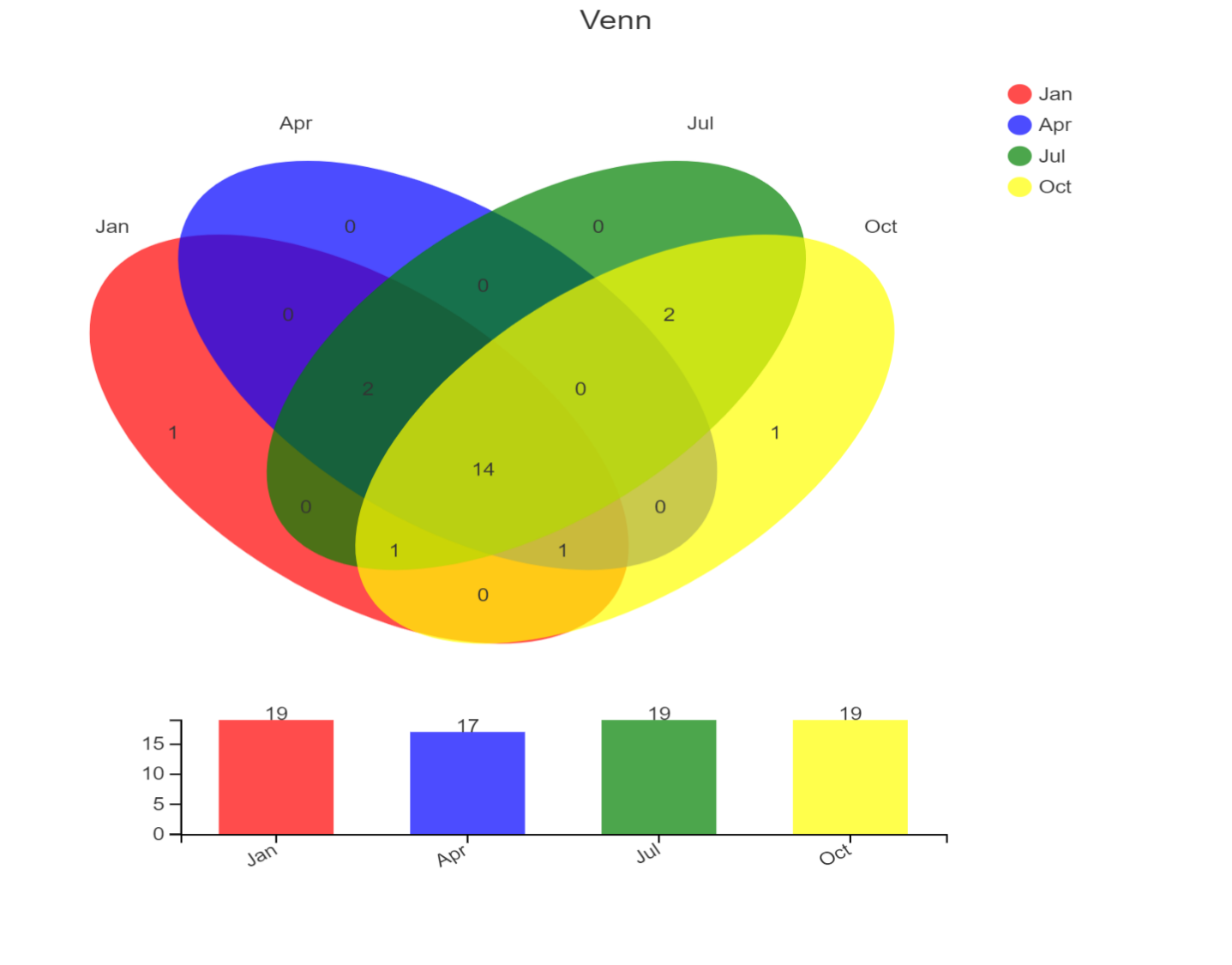

Supplement: Supplementary Figure 3 — Venn diagram showing the OTUs that obtained and overlap among total samples across the four seasons. [file Image_3.TIF]

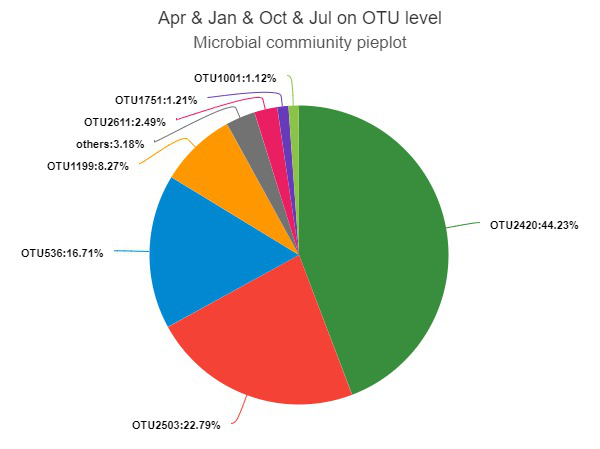

Supplement: Supplementary Figure 4 — Vibrio community structure determined by dominant OTUs counted in the 40 water samples. [file Image_4.TIF]

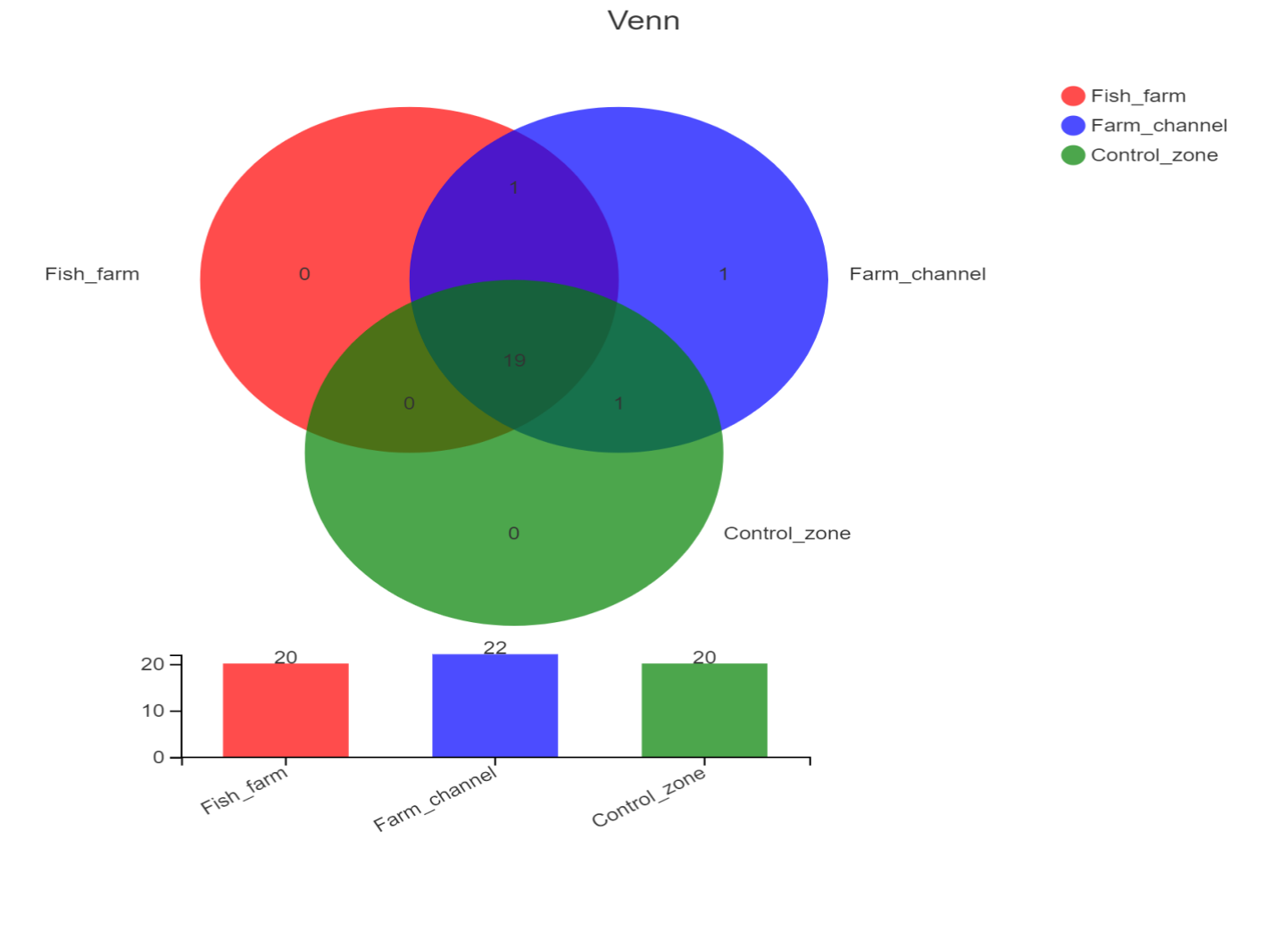

Supplement: Supplementary Figure 5 — Venn diagram showing the OTUs that obtained and overlap among total samples within three different zones. [file Image_5.TIF]

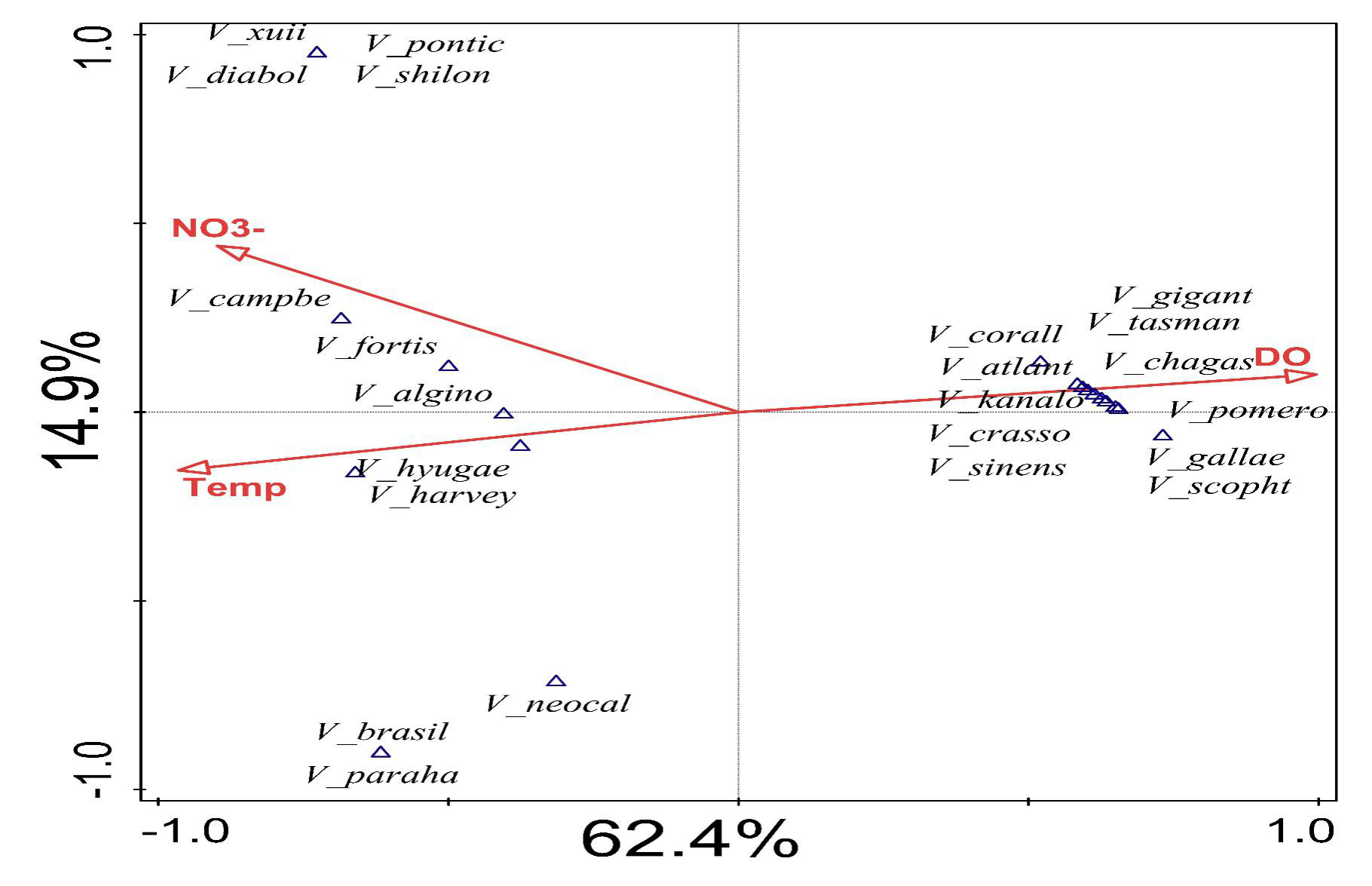

Supplement: Supplementary Figure 6 — RDA biplots for four seasons based on Vibrio isolates data and environmental variables. [file Image_6.TIF]
