# Supplementary material for: Spatiotemporal Dynamics of Vibrio Communities and Abundance in Dongshan Bay, South of China
Source: Front Microbiol. 2020 Nov 26;11:575287. doi: 10.3389/fmicb.2020.575287 (PMC7726330; doi:10.3389/fmicb.2020.575287)
Supplement: Supplementary Table 1 — Environmental parameters of all sampling sites across the four seasons. [file Data_Sheet_1.docx]

Supplementary Material:

Table S1. Environmental parameters of all sampling sites across the four seasons.

| Station | | Season | Temperature(℃) | | pH | DO(mg/L) | Salinity(ppt) | NH_4_^+^ | | PO_4_^3-^ | NO_3_^-^ | NO_2_^-^ |
| --- | --- | --- | --- | --- | --- | --- | --- | --- | --- | --- | --- | --- |
| DY1 | Winter | | | 18.48 | 8.06 | 8.75 | 32.14 | | 0.1124 | 0.2141 | 0.8279 | 0.0166 |
| DY2 | Winter | | | 18.28 | 8.06 | 8.50 | 32.16 | | 0.1596 | 0.1186 | 0.8295 | 0.0106 |
| DY3 | Winter | | | 18.42 | 8.02 | 5.65 | 32.42 | | 0.1596 | 0.1136 | 0.7639 | 0.0075 |
| DY4 | Winter | | | 18.26 | 8.05 | 6.58 | 32.42 | | 0.1278 | 0.1186 | 0.5098 | 0.0189 |
| DS1 | Winter | | | 17.73 | 8.04 | 8.27 | 32.70 | | 0.1278 | 0.1488 | 0.5459 | 0.0113 |
| DS2 | Winter | | | 17.94 | 8.02 | 8.13 | 32.71 | | 0.1287 | 0.1890 | 0.5639 | 0.0528 |
| DS3 | Winter | | | 18.03 | 8.05 | 8.5 | 32.52 | | 0.1596 | 0.2046 | 0.6295 | 0.0106 |
| DS4 | Winter | | | 18.05 | 8.04 | 8.75 | 32.73 | | 0.1677 | 0.2543 | 0.6623 | 0.0121 |
| DS5 | Winter | | | 17.49 | 8.04 | 8.34 | 32.20 | | 0.1557 | 0.1538 | 0.5820 | 0.0038 |
| DS6 | Winter | | | 17.84 | 7.98 | 7.79 | 32.45 | | 0.1514 | 0.1689 | 0.7377 | 0.0339 |
| DY1 | Spring | | | 22.58 | 7.69 | 7.83 | 32.64 | | 0.1660 | 0.1234 | 0.6523 | 0.0515 |
| DY2 | Spring | | | 22.38 | 7.51 | 8.13 | 32.94 | | 0.1760 | 0.1371 | 0.7342 | 0.0619 |
| DY3 | Spring | | | 22.97 | 7.41 | 8.00 | 32.84 | | 0.1002 | 0.1233 | 0.7741 | 0.0821 |
| DY4 | Spring | | | 20.58 | 7.31 | 6.81 | 32.22 | | 0.1122 | 0.1287 | 0.6334 | 0.0633 |
| DS1 | Spring | | | 20.49 | 7.61 | 7.35 | 32.01 | | 0.1165 | 0.1256 | 0.7871 | 0.0762 |
| DS2 | Spring | | | 21.50 | 7.58 | 7.57 | 32.70 | | 0.1176 | 0.1267 | 0.5623 | 0.0777 |
| DS3 | Spring | | | 20.45 | 7.79 | 7.66 | 32.40 | | 0.1201 | 0.1456 | 0.6671 | 0.0914 |
| DS4 | Spring | | | 21.67 | 7.24 | 7.84 | 32.59 | | 0.1104 | 0.1321 | 0.7741 | 0.0531 |
| DS5 | Spring | | | 21.56 | 7.21 | 6.91 | 32.51 | | 0.0918 | 0.1211 | 0.7511 | 0.0613 |
| DS6 | Spring | | | 20.16 | 7.22 | 6.79 | 32.22 | | 0.1010 | 0.1321 | 0.7613 | 0.0679 |
| DY1 | Summer | | | 28.16 | 8.16 | 5.86 | 32.55 | | 0.0962 | 0.1312 | 0.8231 | 0.0971 |
| DY2 | Summer | | | 28.40 | 8.02 | 5.76 | 32.45 | | 0.0977 | 0.1211 | 0.7331 | 0.1214 |
| DY3 | Summer | | | 27.97 | 8.82 | 5.10 | 32.52 | | 0.0967 | 0.1234 | 0.9642 | 0.0824 |
| DY4 | Summer | | | 27.88 | 8.01 | 5.03 | 32.65 | | 0.0923 | 0.1378 | 0.8621 | 0.0974 |
| DS1 | Summer | | | 27.87 | 8.03 | 4.75 | 32.47 | | 0.1003 | 0.1156 | 0.8234 | 0.1028 |
| DS2 | Summer | | | 27.79 | 8.03 | 4.84 | 31.87 | | 0.1130 | 0.1277 | 0.6666 | 0.1138 |
| DS3 | Summer | | | 28.27 | 8.06 | 4.76 | 31.91 | | 0.1986 | 0.1255 | 0.7621 | 0.1024 |
| DS4 | Summer | | | 28.38 | 8.08 | 4.56 | 32.85 | | 0.1976 | 0.1267 | 0.6321 | 0.0917 |
| DS5 | Summer | | | 27.94 | 8.01 | 4.63 | 32.92 | | 0.1678 | 0.1367 | 0.7728 | 0.1017 |
| DS6 | Summer | | | 28.02 | 8.01 | 4.68 | 32.61 | | 0.1646 | 0.1178 | 0.8234 | 0.0974 |
| DY1 | Autumn | | | 26.71 | 7.85 | 5.19 | 32.79 | | 0.1747 | 0.1878 | 0.9132 | 0.1188 |
| DY2 | Autumn | | | 26.6 | 7.88 | 4.95 | 32.83 | | 0.1707 | 0.1825 | 0.8992 | 0.1354 |
| DY3 | Autumn | | | 26.63 | 7.99 | 4.71 | 32.69 | | 0.1922 | 0.1702 | 0.8907 | 0.1261 |
| DY4 | Autumn | | | 26.63 | 7.96 | 4.74 | 32.33 | | 0.1831 | 0.1091 | 0.9246 | 0.1346 |
| DS1 | Autumn | | | 26.6 | 8.01 | 5.08 | 32.12 | | 0.1714 | 0.1722 | 0.8829 | 0.1227 |
| DS2 | Autumn | | | 26.7 | 7.98 | 5.02 | 32.88 | | 0.1663 | 0.1972 | 0.9478 | 0.1394 |
| DS3 | Autumn | | | 26.62 | 7.97 | 5.19 | 32.57 | | 0.1795 | 0.1903 | 0.9010 | 0.1227 |
| DS4 | Autumn | | | 26.57 | 7.97 | 5.01 | 32.05 | | 0.1623 | 0.1091 | 0.9338 | 0.0922 |
| DS5 | Autumn | | | 26.84 | 7.96 | 5.84 | 32.69 | | 0.1025 | 0.1133 | 1.0140 | 0.1294 |
| DS6 | Autumn | | | 26.85 | 7.90 | 5.80 | 32.66 | | 0.1591 | 0.1654 | 0.8724 | 0.1726 |
| * Temperature detection limit 0.01 ℃, accuracy ±0.3+0.005; pH detection limit 0.01, accuracy ±0.1; DO detection limit 0.01 mg/L, accuracy ±0.2 mg/L; salinity detection limit 0.1 ppt, accuracy ±3 ppt | | | | | | | | | | | | |

Table S2. Observed reads and diversity estimates of *Vibrio* spp. based on 97% OTU clusters

| Sample | Raw reads | Clean reads | No. of  OTUs | No. of  *Vibrio*  Reads | No. of  *Vibrio* OTUs | Sample | Raw reads | Clean reads | No. of  OTUs | No. of  *Vibrio*  Reads | No. of  *Vibrio* OTUs |
| --- | --- | --- | --- | --- | --- | --- | --- | --- | --- | --- | --- |
| JanDY1 | 54227 | 43764 | 1368 | 189 | 9 | JulDY1 | 74998 | 46746 | 991 | 121 | 10 |
| JanDY2 | 50035 | 40255 | 1308 | 617 | 10 | JulDY2 | 65475 | 42450 | 1184 | 59 | 10 |
| JanDY3 | 52107 | 42668 | 1148 | 373 | 14 | JulDY3 | 46950 | 35726 | 1158 | 43 | 9 |
| JanDY4 | 43964 | 38151 | 1225 | 165 | 14 | JulDY4 | 45014 | 27607 | 1031 | 67 | 12 |
| JanDS1 | 44915 | 37621 | 1374 | 196 | 9 | JulDS1 | 44834 | 29378 | 983 | 81 | 8 |
| JanDS2 | 48241 | 37336 | 1495 | 155 | 7 | JulDS2 | 57572 | 38654 | 1010 | 64 | 8 |
| JanDS3 | 45796 | 35836 | 1393 | 465 | 11 | JulDS3 | 40169 | 29565 | 832 | 35 | 11 |
| JanDS4 | 48218 | 38734 | 1372 | 206 | 14 | JulDS4 | 56124 | 38236 | 867 | 73 | 15 |
| JanDS5 | 50380 | 37428 | 1374 | 131 | 14 | JulDS5 | 74775 | 51285 | 980 | 42 | 8 |
| JanDS6 | 60288 | 47570 | 1495 | 151 | 15 | JulDS6 | 33762 | 19546 | 733 | 76 | 9 |
| AprDY1 | 53762 | 35677 | 1150 | 204 | 14 | OctDY1 | 48822 | 38291 | 920 | 6012 | 17 |
| AprDY2 | 47919 | 36433 | 1232 | 234 | 15 | OctDY2 | 50830 | 39768 | 1122 | 741 | 15 |
| AprDY3 | 52508 | 38852 | 882 | 63 | 12 | OctDY3 | 51699 | 38280 | 1069 | 1892 | 15 |
| AprDY4 | 59261 | 44083 | 1165 | 114 | 12 | OctDY4 | 60653 | 46522 | 1195 | 324 | 10 |
| AprDS1 | 49450 | 39944 | 1184 | 227 | 9 | OctDS1 | 51511 | 36382 | 1066 | 2120 | 15 |
| AprDS2 | 53296 | 39949 | 1344 | 251 | 11 | OctDS2 | 35248 | 25707 | 819 | 1348 | 11 |
| AprDS3 | 50598 | 35072 | 1057 | 127 | 14 | OctDS3 | 58244 | 43578 | 1052 | 811 | 17 |
| AprDS4 | 45729 | 34667 | 1007 | 45 | 14 | OctDS4 | 42158 | 31822 | 864 | 240 | 13 |
| AprDS5 | 57026 | 44420 | 1002 | 45 | 12 | OctDS5 | 54164 | 37912 | 1119 | 2091 | 16 |
| AprDS6 | 45516 | 38488 | 1141 | 46 | 10 | OctDS6 | 36852 | 29061 | 570 | 2542 | 13 |

Table S3. Classification of obtained 22 *Vibrio* OTUs based on three different database

| OTU | Silva | Ezbiocloud | NCBI | Season obtained from |
| --- | --- | --- | --- | --- |
| OTU607 | s__unclassified_g__*Vibrio* | *Vibrio* sp. (CP045350) | *Vibrio caribbeanicus* | Spring，Summer, Autumn, Winter |
| OTU2405 | s__*Vibrio_fortis* | *Vibrio fortis* |  | Autumn |
| OTU3229 | s__*Vibrio_cholerae*_g__*Vibrio* | *Vibrio mimicus* |  | Winter |
| OTU1812 | s__unclassified_g__*Vibrio* | *Vibrio* sp. (AB470935) | *Vibrio* sp. | Spring，Summer, Autumn, Winter |
| OTU967 | s__unclassified_g__*Vibrio* | *Vibrio maritimus* |  | Winter |
| OTU2420 | s__*Vibrio_fortis* | *Vibrio fortis* |  | Spring，Summer, Autumn, Winter |
| OTU1721 | s__unclassified_g__*Vibrio* | *Vibrio zhanjiangensis* |  | Winter |
| OTU1751 | s__*Vibrio_ponticus* | *Vibrio ponticus* |  | Spring，Summer, Autumn, Winter |
| OTU2461 | s__uncultured_*Vibrio*_sp._g__*Vibrio* | *Vibrio maritimus* |  | Autumn |
| OTU536 | s__*Vibrio_harveyi*_g__*Vibrio* | *Vibrio natriegens* |  | Spring, Summer, Autumn, Winter |
| OTU1001 | s__*Vibrio_renipiscarius* | *Vibrio renipiscarius* |  | Spring，Summer, Autumn, Winter |
| OTU1824 | s__unclassified_g__*Vibrio* | *Vibrio aestivus* |  | Spring，Summer, Autumn |
| OTU1583 | s__unclassified_g__*Vibrio* | *Vibrio echinoideorum* |  | Spring, Autumn, Winter |
| OTU3517 | s__unclassified_g__*Vibrio* | *Vibrio* sp. (AB468986) | *Vibrio* sp. | Summer, Autumn, Winter |
| OTU2611 | s__unclassified_g__*Vibrio* | *Catenococcus thiocycli* | *Vibrio natriegens* | Spring，Summer, Autumn, Winter |
| OTU1776 | s__unclassified_g__*Vibrio* | *Vibrio cortegadensis* |  | Spring, Summer, Autumn, Winter |
| OTU2096 | s__unclassified_g__*Vibrio* | *Vibrio rotiferianus* |  | Spring，Summer, Autumn, Winter |
| OTU2124 | s__unclassified_g__*Vibrio* | *Vibrio hepatarius* |  | Spring, Summer，Autumn, Winter |
| OTU1199 | s__*Vibrio_gigantis* | *Vibrio pomeroyi* |  | Spring，Summer, Autumn, Winter |
| OTU499 | s__unclassified_g__*Vibrio* | *Vibrio caribbeanicus* |  | Spring，Summer, Autumn, Winter |
| OTU2503 | s__*Vibrio_brasiliensis* | *Vibrio neptunius* |  | Spring，Summer, Autumn, Winter |
| OTU489 | s__unclassified_g__*Vibrio* | *Vibrio furnissii* |  | Spring |

Table S4. Classification of obtained 23 *Vibrio* species based on 16S rRNA sequences

| Species | Spring (April) | Summer (July) | Autumn (October) | Winter (January) |
| --- | --- | --- | --- | --- |
| *Vibrio crassostreae* | 13 | 0 | 0 | 21 |
| *Vibrio chagasii* | 2 | 0 | 0 | 6 |
| *Vibrio gigantis* | 3 | 0 | 0 | 2 |
| *Vibrio atlanticus* | 7 | 0 | 0 | 2 |
| *Vibrio gallaecicus* | 1 | 0 | 0 | 2 |
| *Vibrio neocaledonicus* | 0 | 10 | 0 | 1 |
| *Vibrio scophthalmi* | 0 | 0 | 0 | 1 |
| *Vibrio kanaloae* | 1 | 0 | 0 | 1 |
| *Vibrio tasmaniensis* | 4 | 0 | 0 | 1 |
| *Vibrio pomeroyi* | 2 | 0 | 0 | 1 |
| *Vibrio sinensis* | 0 | 0 | 0 | 1 |
| *Vibrio coralliirubri* | 4 | 0 | 0 | 0 |
| *Vibrio hyugaensis* | 2 | 7 | 3 | 0 |
| *Vibrio alginolyticus* | 2 | 7 | 5 | 0 |
| *Vibrio fortis* | 1 | 5 | 8 | 0 |
| *Vibrio harveyi* | 0 | 7 | 3 | 0 |
| *Vibrio parahaemolyticus* | 0 | 3 | 0 | 0 |
| *Vibrio campbellii* | 0 | 2 | 5 | 0 |
| *Vibrio brasiliensis* | 0 | 2 | 0 | 0 |
| *Vibrio shilonii* | 0 | 0 | 3 | 0 |
| *Vibrio diabolicus* | 0 | 0 | 1 | 0 |
| *Vibrio xuii* | 0 | 0 | 1 | 0 |
| *Vibrio ponticus* | 0 | 0 | 1 | 0 |


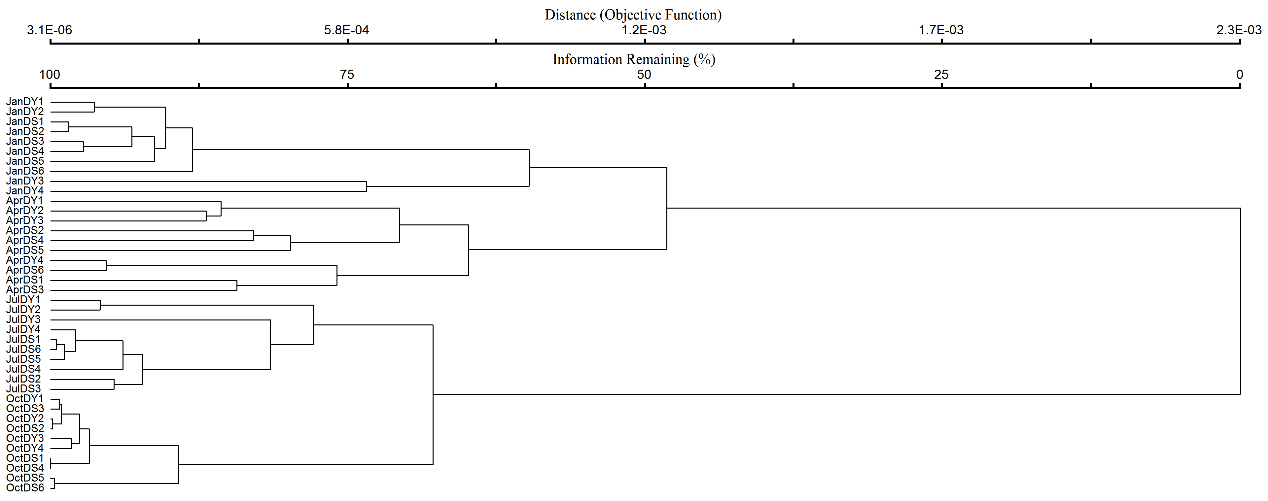


**Fig. S1** Clustering analysis of the normalized environmental parameters using the average cluster method


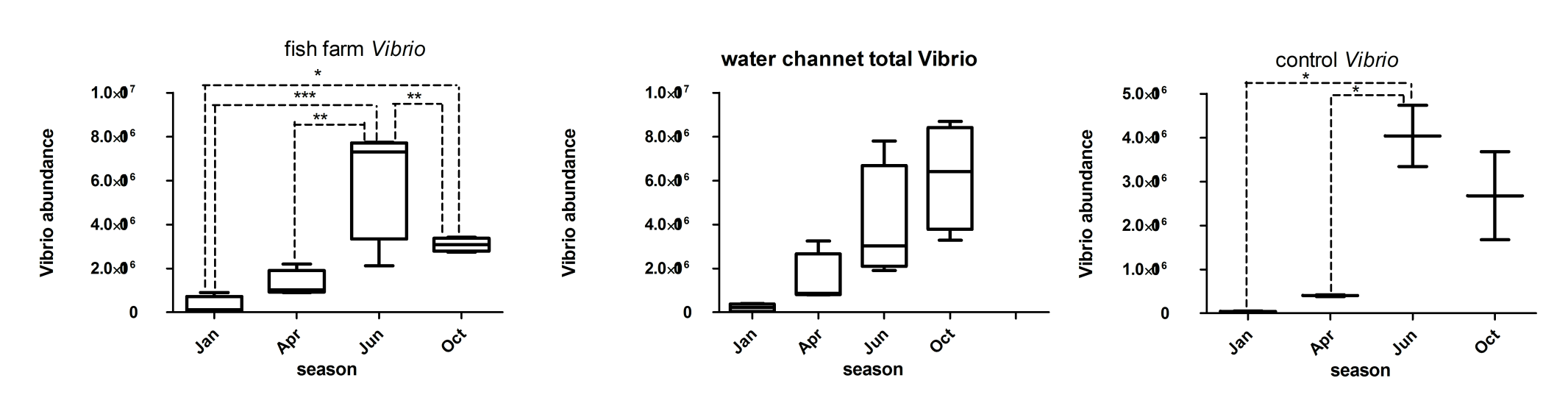


**Fig. S2** *Vibrio* abundance determined by qPCR in the three different sample areas (fish farm, water channel of farm zone and control zone). The asterisks denote significant differences between seasons. *, P <0.05 ** and P <0.01


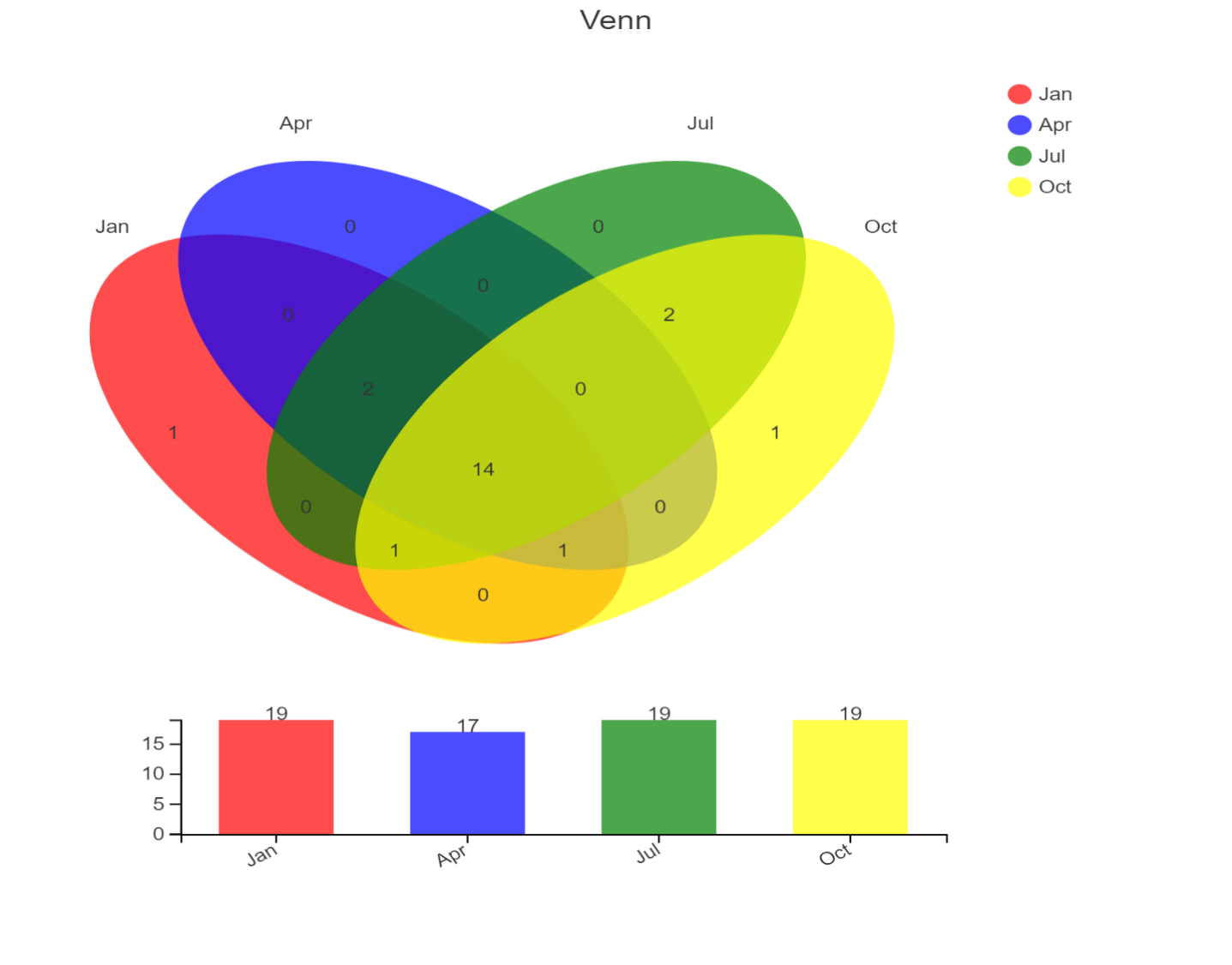


**Fig. S3** Venn diagram showing the OTUs that obtained and overlap among total samples across the four seasons


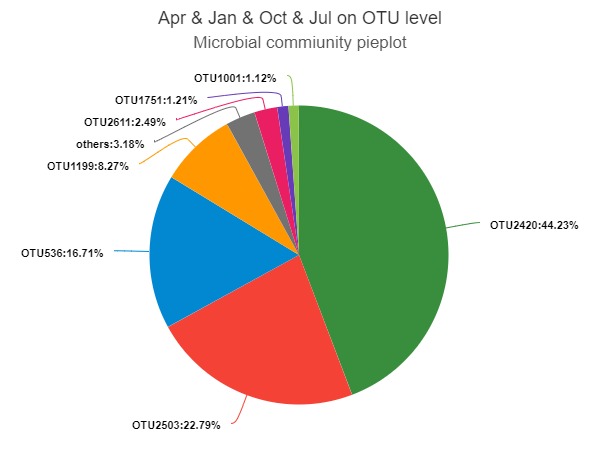


**Fig. S4** *Vibrio* community structure determined by dominant OTUs counted in the 40 water samples


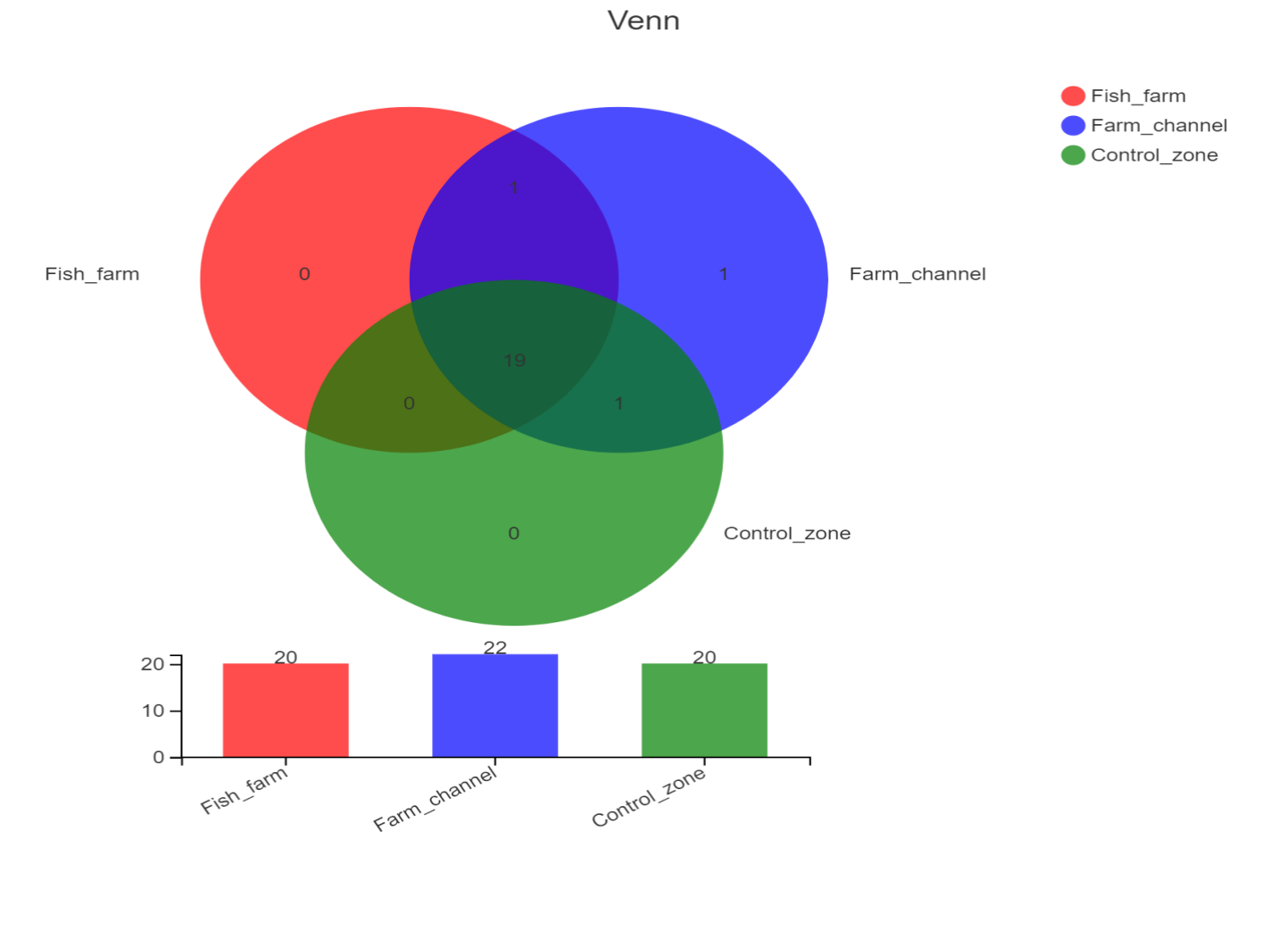


**Fig. S5** Venn diagram showing the OTUs that obtained and overlap among total samples within three different zones


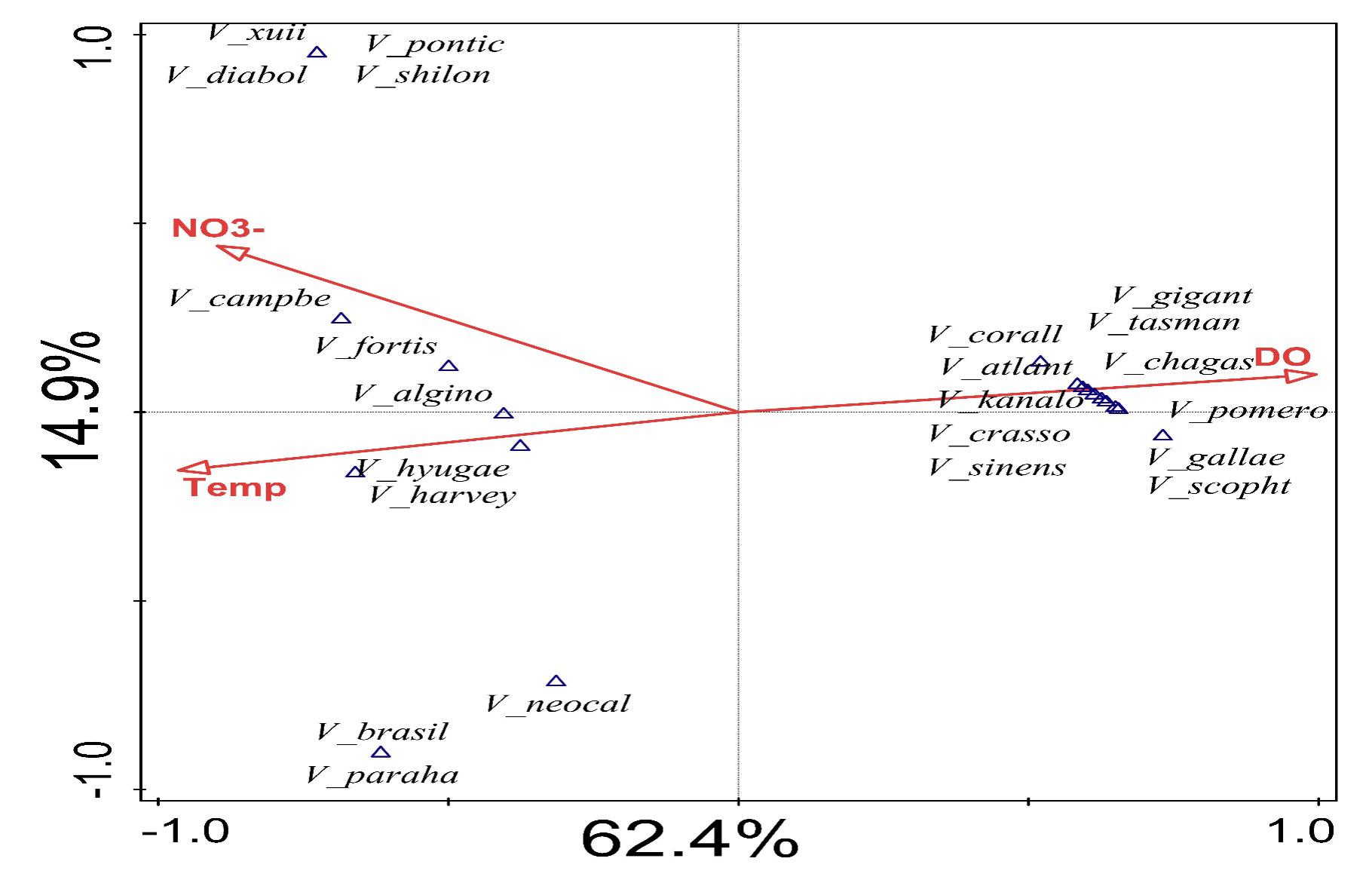


**Fig. S6** RDA biplots for four seasons based on *Vibrio* isolates data and environmental variables

**Influence of environmental variables on microbial community diversity**

The qualified reads were divided into 59 genera, and the abundance of each sample is above 1% (Fig S5). The four most abundance microbes phylum in 40 samples were Proteobacteria (44%~71.5%), Bacteroidota (7.9%~35.8%), Cyanobacteria (3.3%~25.8%) and Actinobacteriota (3.47%~15.6%). The most dominant bacterial genus was Rhodobacteraceae (genus HIMB11) (4.3%~34.3%), followed by NS5_marine_group (1.6%~9.3%) and *Cryomorphaceae* (1.6%~10%) belonging to Flavobacteriales. The highest abundance of Rhodobacteraceae belong to alphaproteobacteria was in DS_JUL_DS3 with proportion reached to 34.3%. Four most genera of gammaproteobacteria were SAR86 clade (order), OM60NOR5 clade (genus), OM43 clade (genus) and *Vibrio*. The most abundance of vibrio presented in the October DS_OCT_DY1, followed by DS_OCT_DS6 and DS_OCT_DS1. While the abundance of photobacteria was less than 1.2% in October and January.


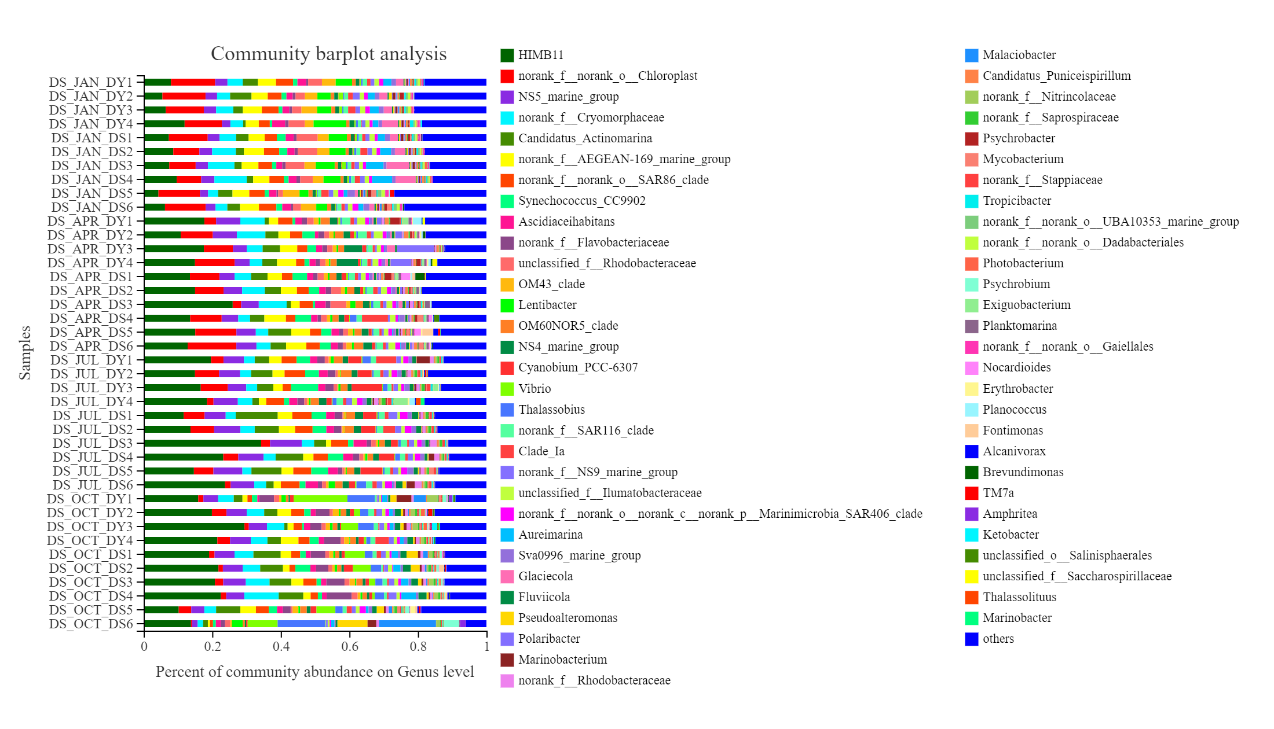


**Fig. S7** Bacteria community compositions at the Genus level across all samples

To summarize the affection of spatial proportion and environmental variables on microbial communities, we applied the redundancy analysis (RDA). The db-RDA showed that approximately 30% of the total variance was explained by the constrained matrix, and it clearly segregated the samples with seasons (FigS6.). It was interesting to note that samples in October were more discrete than samples in the other three seasons. While samples in January could be seen within the same cluster. The strongly significant environmental factors were DO (R^2^=0.78, P =0.001), Nitrite (R^2^=0.74, P =0.001), Temperature (R^2^=0.70, P =0.001), pH (R^2^=0.67, P =0.001) and Nitrate (R^2^=0.51, P =0.001). Our results show that one main point, which is that the difference in microbiota composition is mainly due to dissolved oxygen.


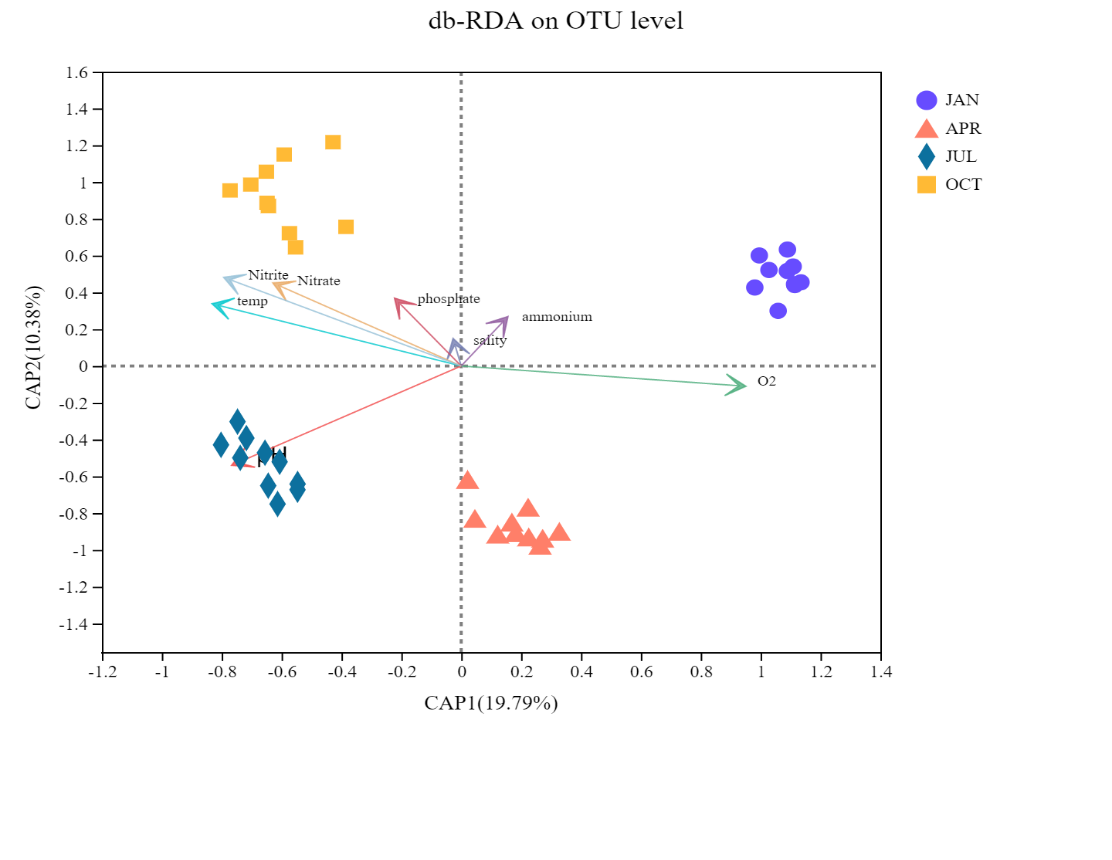


**Fig. S8** Distance-based redundancy analysis (db-RDA) of all detected operational taxonomic units (OTUs) and environmental variables
